# Assessing heat tolerance in potatoes: Responses to stressful Texas field locations and controlled contrasting greenhouse conditions

**DOI:** 10.3389/fpls.2024.1364244

**Published:** 2024-05-13

**Authors:** Sanjeev Gautam, Douglas C. Scheuring, Jeffrey W. Koym, M. Isabel Vales

**Affiliations:** ^1^ Department of Horticultural Sciences, Texas A&M University, College Station, TX, United States; ^2^ Texas A&M AgriLife Research and Extension Center, Lubbock, TX, United States

**Keywords:** high temperature, heat stress, climate change, marketable yield, tuber defects

## Abstract

In recent years, heat stress has affected potato production more frequently, resulting in lower marketable yields and reduced tuber quality. In order to develop heat-tolerant potatoes, it is necessary to select under heat-stress conditions and consider traits affected by heat stress. The Texas A&M Potato Breeding Program has selected potatoes under high-temperature stress for several decades. Ten potato cultivars, representing heat tolerant and sensitive clones based on past performance in Texas, were included in field trials for three years at the two main locations used by the Texas Breeding Program (Dalhart and Springlake, TX) to assess if the Texas field locations are suitable for heat tolerance screening. Both locations were confirmed as appropriate for heat stress screening. However, Springlake was a more stressful location since it had significantly lower yields of marketable tubers and increased percentages of tuber defects. Planting time did not have a significant effect at the most stressful location. The same ten potato clones were included in greenhouse experiments with contrasting temperatures (normal versus heat stress). There was confirmation that heat stress conditions resulted in significantly lower marketable yields, specific gravity, dormancy, and significantly higher percentages of tuber defects; however, significant differences existed between potato clones. Under heat stress conditions, Russet Burbank had a high percent of tubers with external defects, whereas Atlantic showed the highest percentage of internal defects (mainly internal heat necrosis). Vanguard Russet produced the highest marketable yield while maintaining a low percentage of external and internal defects. Russet Burbank and Atlantic were heat-sensitive controls for external and internal tuber defects, respectively. In contrast, Vanguard Russet can be used as a reliable heat-tolerant control. Including appropriate controls in heat stress studies will help identify clones with heat tolerance.

## 1 Introduction

The human population is projected to reach 9.8 billion by 2050 (United Nations, 2017), requiring 100 to 110% more food in 2050 than in 2005 (Tilman et al., 2011). However, a decrease in crop yields due to climate change (Lobell and Field, 2007; Zhao et al., 2022) poses a significant challenge in ensuring adequate food production for a growing population (Tito et al., 2018). Earth’s average temperature is predicted to rise by 1.1-6.4°C due to increased greenhouse gases during the next century (Yamori et al., 2014). Increasing temperatures are anticipated to hinder advancements in addressing food insecurity by diminishing agricultural yields in the upcoming decades (Kroeger, 2023). The rise in temperature demands crops to grow and produce well at temperatures above their optimal. Potatoes are among the many crops affected by increased temperatures and climate change.

Potato is a cool season crop that produces tubers (the edible part, underground modified stems used for nutrient storage and propagation). Temperature and photoperiod regulate potato tuberization and yield (Dutt et al., 2017). However, temperature has been cited as the single most crucial uncontrollable factor affecting the growth and yield of potatoes (Levy and Veilleux, 2007). Potato plants can tolerate day temperatures of about 32°C without significant loss in total biomass production (Minhas et al., 2006); however, a temperature of 25°C has been considered optimum for vegetative growth and 24°C for photosynthesis (Timlin et al., 2006). The most temperature-critical growth stages in potatoes are tuber initiation and bulking. Night temperatures above 18°C reduce tuberization, whereas temperatures beyond 25°C (Bushnell, 1925) may result in no tuberization. Several reports have shown tuber yield and quality deterioration due to heat stress (temperatures beyond the upper limit of the optimum tuberization - 20°C) (Lafta and Lorenzen, 1995; Hancock et al., 2014; Aien et al., 2017; Paul et al., 2017; Obiero et al., 2019). Heat stress damage is more apparent in tubers than in shoots (Lafta and Lorenzen, 1995). Moreover, the growth stage at which heat stress occurs has an even more significant implication on the cultivar’s response to high temperatures. The effect of heat stress is more pronounced in the early stages of tuber formation than in later stages. Heat stress at early stages results in drastic tuber yield reductions (Rykaczewska, 2013) and at later stages leads to quality deterioration (Kim et al., 2017). The effect of heat stress was more deleterious under long days than under short days; complete inhibition of tuber formation in the variety Désirée was observed when grown at 16:8 h (day: night) photoperiod and 32/22°C (day/night) temperature (Wolf et al., 1990).

Heat stress impacts both potato yield and tuber quality (external as well as internal), causing deformities like elongation, bottlenecks, second-growth, chain tuberization, gemmations, and knobs during tuber bulking (Rykaczewska, 2013). Assessment of tubers’ secondary growth and physiological defects needs to be incorporated into heat tolerance studies of potatoes since total yield alone may not be the only indicator of tolerance (Rykaczewska, 2015). High temperatures during plant growth often shorten the dormancy period of potato tubers (Wurr, 1992; Levy and Veilleux, 2007; Struik et al, 2007). The effects of high temperatures on potatoes are aggravated when coupled with water deficiency, resulting in significant yield loss (Levy, 1986). Rykaczewska, 2015 reported that combined heat and drought stress reduced the yield of a sensitive cultivar by over 50%, whereas the more tolerant variety experienced a yield reduction of about 25%.

The effects of heat stress vary according to genotype, occurrence time, duration, and intensity of high temperatures. Potato cultivars differ in their responses to heat stress (Levy, 1986; Ghosh et al., 2000; Ahn et al., 2003). Popular varieties like Russet Burbank, Atlantic, and Yukon Gold are known to be sensitive to internal heat necrosis or internal brown spots. The frequency and severity of such defects increase when they experience high day and night temperatures early in the growing season, combined with low rainfall (Yencho et al., 2008). The tolerance of potatoes towards heat stress is explained by various biochemical and transcriptional factors differing in genotypes (Morpurgo and Ortiz, 1988).

Potato-growing regions in the world are changing (Jennings et al., 2020; Devaux et al., 2021). While new areas are added, traditional regions face challenges in growing potatoes. To meet the current production level, around 12.5% of the current potato production climate (43 million hectares) is required to be shifted to newer regions (Fumia et al., 2022). However, developing heat-tolerant potato varieties is the practical solution to sustain and expand production, aiding food security in the face of climate change and global warming (Hijmans, 2003; Pradel et al., 2019; Devaux et al., 2021). Heat-tolerant varieties are projected to increase the potential yields by more than 5% in most areas and 10% in tropical regions and parts of the USA and Canada (Hijmans, 2003). Manipulating planting dates and developing tolerant varieties are crucial strategies, as shown in a simulation study (Naz et al., 2022).

Of the 156 countries worldwide that plant potatoes (FAO, 2019), heat stress is reported in various places, including tropical highlands. Lavras in Brazil (Lambert et al., 2006); San Ramón in Peru (Khan et al., 2015; Benavides et al., 2017; Raymundo et al., 2018; Muñoa et al., 2022); Jalandhar in India (Bhardwaj et al., 2022), Western Australia in Australia (Obiero et al., 2022) are considered heat stress sites for the selection of heat-tolerant potatoes. Some unexpected areas, like Ontario, Canada, have also been reported to experience heat during the potato growing season. Potato yield decreased by 17.2% in 2016 compared with the production in 2015 in Ontario, Canada, due to extremely high temperatures during the 2016 summer (Tang et al., 2018). High-temperature stress will be more common in the future (Battisti and Naylor, 2009; Monneveux et al., 2014; Lehretz et al., 2021), emphasizing the need for developing heat-tolerant varieties (Bashir et al., 2023). Screening potato genotypes for heat tolerance under appropriate conditions is a reliable method to select clones enriched with favorable traits/genes that allow them to overcome heat stress. The Texas A&M Potato Breeding Program has been selecting potatoes under high-temperature stress (>30°C) for several decades at two main locations in the Panhandle region of Texas, Springlake and Dalhart, and has released several potato clones that could be considered heat-tolerant.

This study was performed to 1) evaluate if Texas field locations used by the Texas A&M Potato Breeding Program are suitable for heat stress screening (objective 1), 2) assess if planting time at the most stressful location should be modified to improve heat stress screening (objective 2), and 3) identify tuber traits affected by heat stress under field conditions and compare them with those observed under controlled greenhouse conditions (objective 3). The goal is to identify appropriate settings (field locations, planting dates, contrasting greenhouse conditions) to evaluate potato clones for heat stress and to identify reliable heat tolerant and sensitive checks for future potato heat tolerance studies. Heat-tolerant potato clones are desired to cope with the future global warming scenario and extend potato production to warmer areas.

## 2 Materials and methods

### 2.1 Plant materials

Ten potato genotypes were used in this study: six varieties released by the Texas A&M Potato Breeding Program and four reference/check varieties. The reference varieties included a processing chipper (Atlantic) and a processing French fry russet (Russet Burbank) widely planted in the USA; a fresh market russet (Russet Norkotah); and a yellow flesh yellow skin cultivar (Yukon Gold). Plant maturities ranged from early to late (**Table 1**).

**Table 1:** Tuber characteristics and plant maturity of the ten potato clones planted in Springlake and Dalhart, Texas, during 2019, 2020, and 2021.

Clone	Code	Tuber skin type	Tuber flesh color	Market class	Plant maturity	References
Atlantic	AT	Smooth (scaly net)	White	Processing (Chipping)	Medium	Webb et al., 1978
COTX09022-3RuRE/Y	CO	Russet	Yellow	Dual	Early-Medium	Vales et al., 2020
Reveille Russet	RR	Russet	White	Fresh	Late	Miller et al., 2018
Russet Burbank	RB	Russet	White	Processing (French fries)	Medium	Brown, 2015
Russet Norkotah	RN	Russet	White	Fresh	Medium	Johansen et al., 1988
Russet Norkotah 278	RN278	Russet	White	Fresh	Medium	Miller et al., 1999
Russet Norkotah 296	RN296	Russet	White	Fresh	Medium	Miller et al., 1999
Sierra Gold™	SG	Russet	Yellow	Fresh	Early	Miller et al., 2005
Vanguard Russet	VR	Russet	White	Fresh	Medium-late	Vales et al., 2022
Yukon Gold	YG	Smooth	Yellow	Fresh	Early	Johnston and Rowberry, 1981

### 2.2 Experimental design and analysis

A randomized complete block design was used for experiments linked to objective 1. The experiments consisted of two locations: Springlake (34°6′N, 102°19′W) and Dalhart (35˚58′15′′N, 102˚44′36″W). Trials were conducted at each location, with four replications in 2019 and three replications in 2020 and 2021. Planting and harvest times and crop duration are provided in **Supplementary Table 1.**

**Figure 1:**
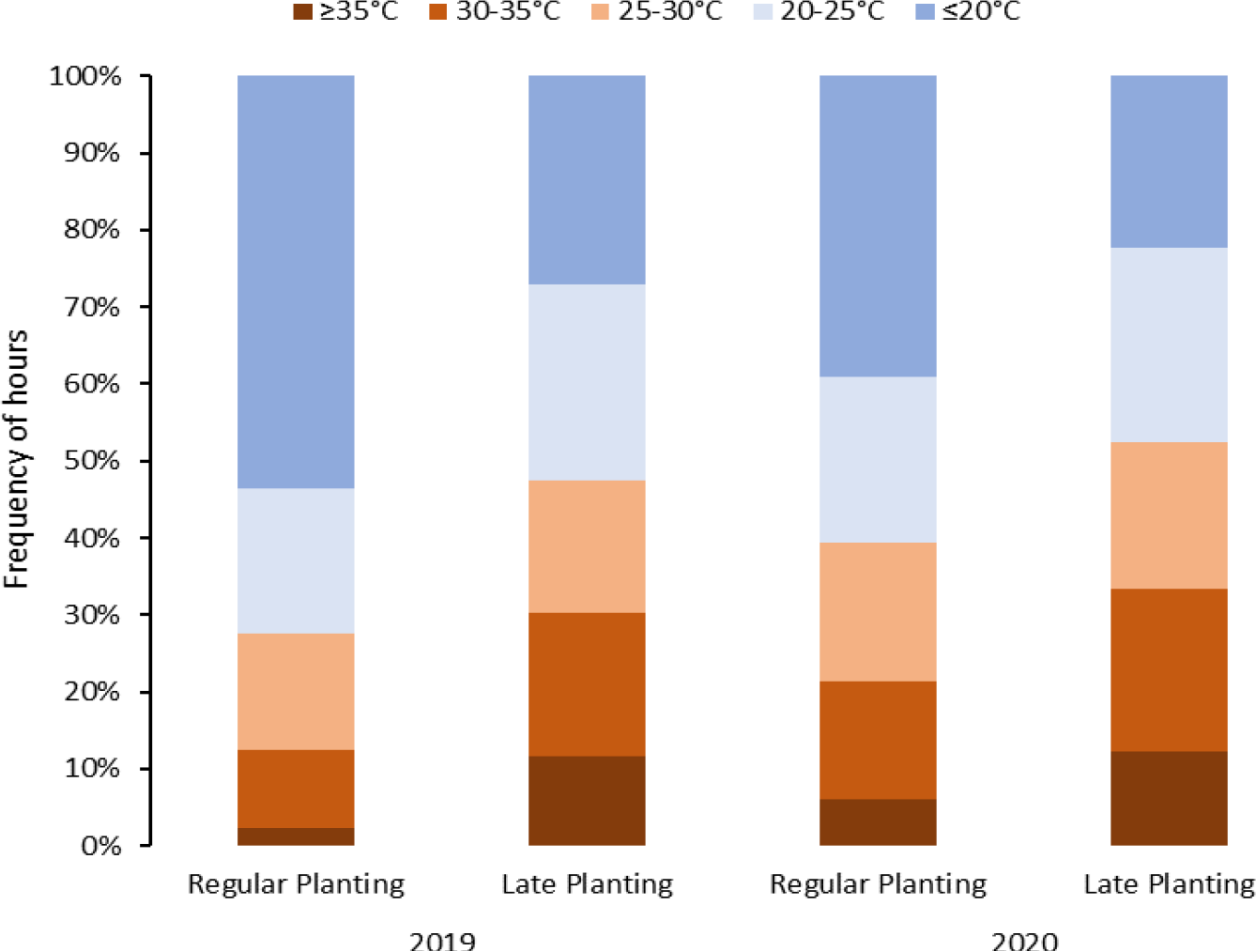
Temperature distribution during the crop period under different planting dates (normal *vs.* late) for two years (2019 and 2020) in Springlake, Texas.

A split-plot design was used for experiments included in objective 2 in Springlake. The experiments consisted of two different planting times (main plot factor): regular planting practiced by growers and late planting to capture higher temperatures during crop growth (**Figure 1**). Late or delayed planting can represent an alternative option mimicking higher-temperature stress treatment during the crop cycle for heat stress. The International Potato Center (CIP) evaluates its breeding population in field conditions with late planting for heat stress (Monneveux et al., 2014). Ten genotypes (subplot factor) were planted with four replications. Planting and harvest times and crop duration are provided in **Supplementary Table 2.**

In 2020 and 2021, factorial block designs were used for experiments related to objective 3. Ten clones were planted in two greenhouses near Snook, TX (30°31′N, 96°25′W), with one greenhouse subjected to normal conditions and the other to heat stress. Each clone and condition had four replications, consisting of three plants per replication in 11.4-L pots. Greenhouse temperatures were set at 25/15°C day/night for 30 days post-planting, then one greenhouse remained at 25/15°C while the other was adjusted to 35/25°C for the remainder of the experiment. GROWCOM systems (Microgrow, Temecula, CA, USA) were used for temperature control, with evaporative cooling and vented forced-air propane heaters. Vines were killed after approximately 90 days, and tubers were left in pots for ten days before harvesting on May 27, 2020, and June 3, 2021 (**Supplementary Table 3**).

### 2.3 Traits measured

After harvesting, the tubers were graded, counted, and weighed according to standard size groups used by the breeding programs (Vales et al., 2020).

#### 2.3.1 Total and marketable yield

The tubers with defects or culls (CU) (heat sprouts, chain tubers, knobs including irregular shapes and secondary growths, growth cracks, green heads, soft rots, insect damaged, cut tubers) and small tubers (<114 g) were categorized as non-marketable tubers, whereas defect-free tubers above 114 g were regarded as marketable tubers (US). Total yield (TY) was categorized as marketable (US), culls (CU), and small tubers (SM); their percentage as the percentage of marketable tubers (PUS) and percentage of cull tubers (PCU), respectively.

#### 2.3.2 Percentage tubers with external and internal defects

For trials related to objective 2, the cull tubers (CU) were further categorized into their prominent external tuber defects (heat sprouts, chain tubers, knobs, and growth cracks) separated from defects such as greenheads, insect-damaged, cut tubers, and soft rots. The count of the tubers in each defect category was recorded and later expressed as a percentage of the total tubers with external defects (PTED). For internal tuber defects, ten tubers were cut from the bud end to the stem end. The presence of internal defects (hollow heart, black spot, vascular discoloration, and internal heat necrosis) was recorded per tuber and expressed as a percentage of internal defects (PID).

#### 2.3.3 Average tuber number and tuber weight

The average tuber number (ATN) was obtained by dividing the total tuber count by the plant count 60 days after planting. Average tuber weight (ATW) was obtained by dividing the total tuber weight by the total tuber number in the plot.

#### 2.3.4 Specific gravity and dry matter

The specific gravity (SG) of tubers was evaluated by comparing the weight of marketable grade tubers (~1 kg) in the air and water using the formula: specific gravity = [weight in air/(weight in the air - weight in water)]. The potatoes were cut longitudinally from stem to bud end in quarters; one quarter from each tuber was chopped and mixed thoroughly, and 15 g of fresh tuber samples were stored in 50 mL Falcon tubes. To calculate tuber dry matter (DM), each sample, fresh weight (FW), was obtained first, then immediately frozen at -20°C and later transferred to - 80°C for a few days before freeze-drying. The samples were freeze-dried (LABCONCO, FreeZone console freeze dryer 6L −50°C Series, Kansas City, MO, USA) with a collector temperature of (-50°C) and vacuum pressure of 0.21 mbar for five days. Freeze-dried samples were weighed to obtain dry weight (DW), and DM was calculated using the formula DM% = (DW/FW) *100. Tuber dry matter (DM%) was used to calculate total tuber dry matter, which was used to calculate the harvest index.

#### 2.3.5 Dormancy

Tuber dormancy (DOR) was assessed in 2020 and 2021 using uniform-sized tubers (114g to 510g) from field and greenhouse experiments. Four tubers per trial were placed in mesh bags and stored in a darkroom at room temperature (20 ± 2°C, 60% RH). Dormancy was considered broken when three out of four tubers showed signs of sprouting (peeping or 2-3 mm sprouts visible). The dormancy period was calculated as the days from vine kill to peeping.

#### 2.3.6 Additional traits

Additional traits like plant height (PH), leaf area (LAI), above-ground dry matter (ADM), and harvest index (HI) were collected from greenhouse experiments. Plant height was taken before vine kill at 90 days after planting. Leaf area was measured on the 4^th^-5^th^ fully formed leaf from the top, taken 90 days after planting before cutting the vines. Three leaf samples per clone per replication were measured for leaf area using LI-3100C leaf area meter (LI-COR Biosciences, Lincoln, NE, USA). Vines were cut 90 days after planting and dried in brown paper bags in a Tru-Temp Oven (Hotpack Corporation, Philadelphia, PA 19154, USA) set at 100°C. Harvest index (HI) was calculated as HI= Total tuber dry matter/(above ground matter + total tuber dry matter).

### 2.4 Temperature data

**Figure 2:**
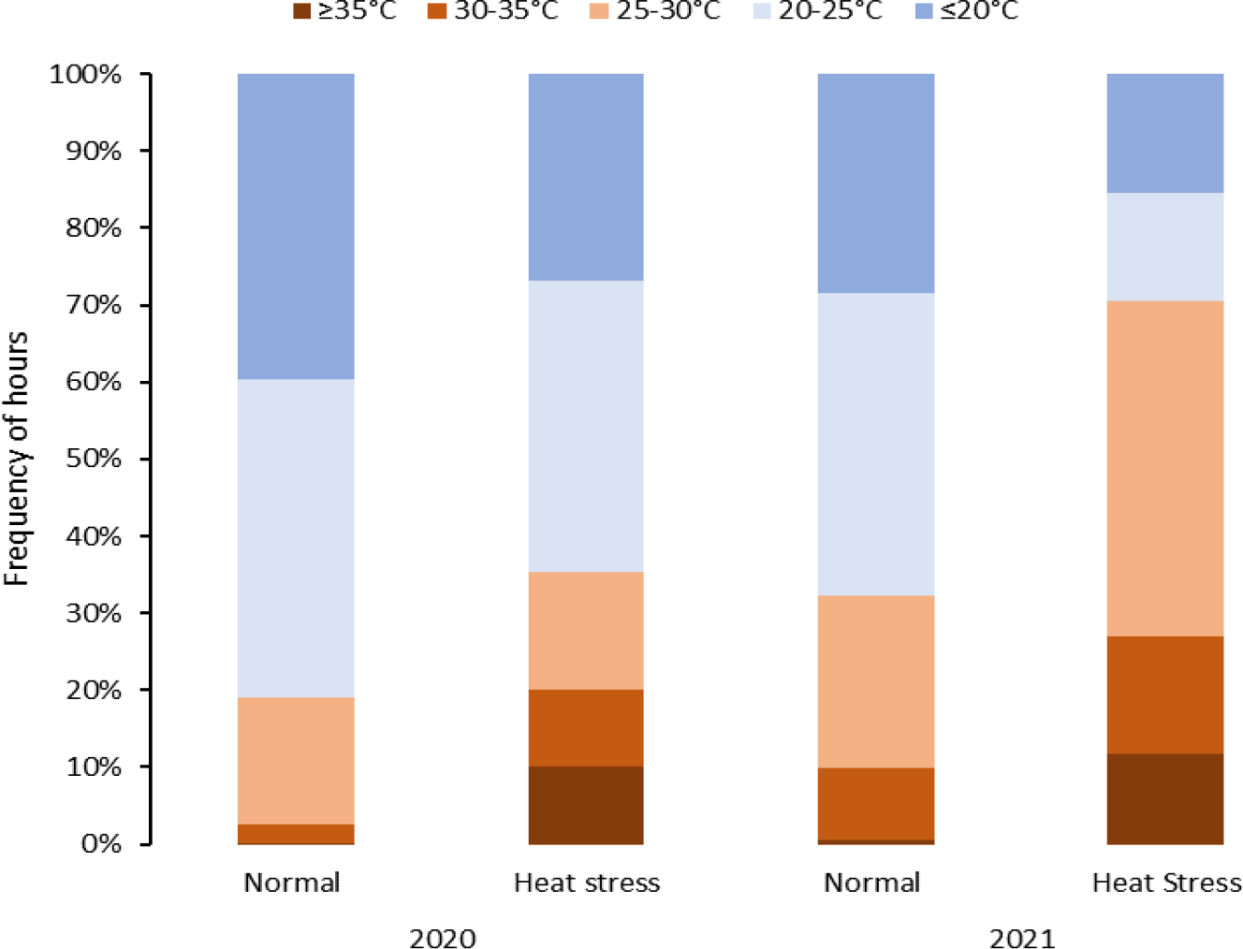
Temperature distribution during the crop growing period affected by heat stress (temperatures > 25°C) in greenhouse experiments under normal and heat stress conditions (2020 and 2021).

For the experiments related to objective 1, temperature data was retrieved from NOAA (https://www.ncdc.noaa.gov/cdo-web/, accessed on December 12, 2022) for both Texas locations, including maximum and minimum temperatures/day during the crop period. The temperature conditions at each location are summarized in **Table 2** and discussed in our previous work (Gautam et al., 2024). For objectives 2 and 3 trials, the temperatures were monitored using the EL-USB-1 temperature loggers (Dataq Instruments Inc., Ohio, USA). The data-loggers were kept one meter above ground to record the air temperature of the field. Temperatures were recorded at intervals of an hour, and the temperatures were categorized into intervals (<20, 20-25, 25-30, 30-35 and >35°C) (**Figures 1**, **2**).

**Table 2:** Temperature conditions at two potato growing locations in Texas - Springlake and Dalhart - for three years (2019, 2020, and 2021) (based on NOAA)*.

Temperature conditions defining stress	2019		2020		2021	
	Springlake	Dalhart	Springlake	Dalhart	Springlake	Dalhart
Stressful days (Max >35°C & min >20°C)	0	5	8	5	7	1
Stressful days (Max >30°C & min >20°C)	0	7	11	5	7	1
Stressful days (Max >25°C & min >20°C)	0	7	11	5	7	1

*NOAA, National Oceanic Atmospheric Administration.

### 2.5 Statistical analysis

Data were entered in Microsoft Excel, and analysis of variance was done using a mixed model with JMP pro17(SAS Institute Inc, 2022). For experiments related to objective 1, analysis of variance was conducted with replication as random effects; clones and locations as fixed effects. Since the test of homogeneity of variance showed heterogeneous variances between the years, the experiments were analyzed separately by year. For trials related to objective 2, analyses of variance was conducted, treating years and replications (nested within years) as random effects but clone and planting dates as fixed effects. For greenhouse experiments (objective 3), statistical analysis was employed, considering clones and conditions as fixed effects and replications as random. Mean comparisons were conducted using Tukey’s HSD in all the experiments.

Relationships between environments and the performance of clones were evaluated using the GGE Biplot method (Yan and Tinker, 2006) with “metan” package (Olivoto and Lúcio, 2020) in the R environment (R Core Team, 2020). Multi-trait stability index (MTSI) (Olivoto et al., 2019) was used to select the top-performing clones under field conditions (objectives 1 and 2) and greenhouse (normal *vs.* heat stress) trials (objective 3). MTSI weighs desirable traits positively and undesirable traits negatively.

## 3 Results

### 3.1 Temperature conditions

Springlake and Dalhart experienced high temperatures throughout the crop season (**Table 2**). In Springlake, temperatures steadily rose until harvest, while in Dalhart, temperatures peaked 60-70 days after planting and gradually declined (Gautam et al., 2024). Photoperiod followed a similar trend: in Dalhart, day length peaked at 50-60 days (14 hours 36 minutes) then sharply decreased to 12 hours, while in Springlake, it reached 14 hours 24 minutes near harvest (**Supplementary Figure 1**). Thus, temperatures kept increasing during the season at Springlake and did not benefit from photoperiod reduction that favors tuberization.

Considering above 25°C as a heat stress condition, potato crops in Springlake experienced heat stress conditions for approximately 28% to 38% of the crop period in regular planting and 45% and 48% in late planting (**Figure 1**). If we consider temperatures above 20°C stressful, Springlake will experience around 46% and 57% of crop period in regular planting but about 68% and 71% in late planting. According to the study, late planting was observed to constantly experience high temperatures, almost 40% in 24 hours. The stressful hourly temperatures continued increasing until harvest during the crop period for regular planting. However, hourly stressful temperatures remained relatively constant throughout the crop period for late planting.

Greenhouses set for normal temperatures (in 2020 and 2021) experienced milder temperatures than those set for heat stress (**Figure 2**, **Table 3**). Temperatures below 25°C in the greenhouse for normal temperatures represented 80% and 67% of the crop period in 2020 and 2021, respectively. On the other hand, the greenhouses set for heat stress endured temperatures above 25°C for 36% and 70% of the crop period in 2020 and 2021, respectively. Considering the number of stressful days for the greenhouses set normal and heat stress conditions, there were more stressful days in 2021 than in 2020 as external weather conditions affected greenhouse temperature control, and the controls worked better in 2021 for heating. However, cooling the greenhouses to obtain normal temperatures was challenging when it was hot and humid outside since the evaporative cooling systems had difficulty operating properly. In any case, the normal and high-temperature conditions were contrasting in both years but more differentiated in 2021.

**Table 3:** Temperature conditions for two different growing conditions (normal *vs.* heat stress) in greenhouses for two years (2020 and 2021).

Temperature conditions defining stress	2020		2021	
	Normal	Heat stress	Normal	Heat stress
Stressful days with day >35°C and night >25°C	0	2	0	25
Stressful days with day >30°C and night >25°C	1	2	0	26
Stressful days with day >35°C and night >20°C	1	19	9	55
Stressful days with day >30°C and night >20°C	11	24	34	66

### 3.2 Analysis of variance of traits

The test for fixed effects on experiments related to objective 1 (effect of location) showed significant location by clone interaction for most traits measured, except total yield in 2019, average tuber number per plant and specific gravity in the year 2020, and percent tubers with internal defects in the year 2021 (**Supplementary Table 4**). Although the interaction between location and clone was statistically significant, location explained most variation for most traits evaluated (**Supplementary Figure 2**).

For trials associated with objective 2 (effect of planting date) involving planting dates in Springlake (the most stressful location), the test on fixed effects indicated no significant differences for planting date. Still, there were significant differences between clones for most traits, except total yield and average tuber number (**Supplementary Table 5**). Clones explained the largest phenotypic variation (~30%) for most traits, except total yield and average tuber number per plant. Planting date and year explained considerable variation in total yield and average tuber per plant (**Supplementary Figure 3**).

Considering the greenhouse trials (objective 3), the test for fixed effects indicated a significant effect of growing conditions (normal *vs.* heat stress) for all traits in 2021 and only four traits in 2020. There was a significant condition by clone interaction for all traits measured except dormancy in both the years, plant height, aerial dry matter, and harvest index in 2021 (**Supplementary Table 6**). Although the interaction between condition and clone was statistically significant, clones explained most of the variation (~30%) for most traits **(Supplementary Figure 4**).

#### 3.2.1 Total yield

**Figure 3:**
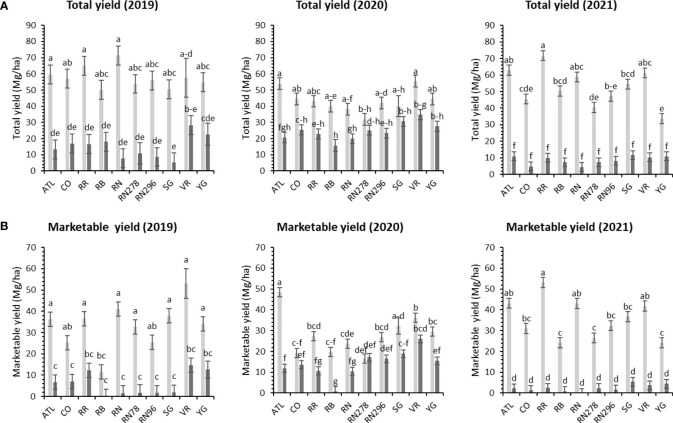
Least squares mean of **(A)** total yield, and **(B)** marketable yield in ten potato clones (x-axis) grown at two different locations (Dalhart - light grey bars and Springlake - dark grey bars) of TX, in 2019, 2020 and 2021. Vertical bars represent mean ± SE. Bars with different letters differ significantly at p<0.05 (Tukey’s HSD).

Significant yield differences existed between the Springlake and Dalhart locations for total yield (TY) in all three years (2019, 2020, and 2021), with Dalhart consistently yielding higher. Clones were significantly different in 2020 and 2021 but not in 2019 (mainly due to high total yields and no significant differences between clones in Dalhart) (**Supplementary Table 4**). On average, 67% total yield reduction was observed in Springlake (range: 8.6 Mg/ha - 24.6 Mg/ha) *vs.* Dalhart (range: 43.3 Mg/ha to 57.5 Mg/ha) (**Table 4**, **Figure 3**). Location explained more than 50% of the phenotypic variation (**Supplementary Figure 2**) in total yield in all the years tested. The highest-yielding genotypes in Dalhart were Vanguard Russet (55.4 Mg/ha) along with Atlantic (53.9 Mg/ha) in 2020 and Reveille Russet (71.7 Mg/ha) in 2021. The lowest yielding genotypes in Springlake were Russet Burbank in 2020 (15.7 Mg/ha) and 2021 (7.4 Mg/ha), along with Reveille Russet (10 Mg/ha), Russet Norkotah (4.5 Mg/ha) and its strains (7.7 Mg/ha and 8.2 Mg/ha) in 2021 (**Figure 3**).

**Table 4:** Least squares means of different traits on experiments for assessing the effect of location on yield and quality traits of ten selected potatoes grown in Texas during the years 2019-2021.

Location	Year	Total yield	Marketable yield	Percent tubers with internal defects	Average tuber number	Average tuber weight	Specific gravity	Dormancy
		Mg/ha	Mg/ha	%	no/plant	g/tuber		Days
**Dalhart**	**2019**	57.5	33.4	16.2	8.4	140.5	1.064	
	**2020**	43.3	27.9	16.4	4.9	166.9	1.066	96
	**2021**	52.6	35.6	16.7	5.9	168.7	1.066	110
***Average Dalhart***		***51.1***	***32.3***	***16.4***	***6.4***	***158.7***	***1.065***	***103***
**Springlake**	**2019**	14.9	6.1	22.9	3.2	100.4	1.058	
	**2020**	24.6	14.2	6.6	4.5	114.8	1.068	62
	**2021**	8.6	2.5	5.0	3.7	59.0	1.067	76
***Average Springlake***		***16.0***	***7.6***	***11.5***	***3.8***	***91.4***	***1.064***	***69***
**Change %**	**2019**	-74.1	-81.7	41.4	-61.9	-28.5	-0.6	
	**2020**	-43.2	-49.1	-59.8	-8.2	-31.2	0.2	-35.4
	**2021**	-83.7	-93.0	-70.1	-37.3	-65.0	0.1	-30.9
***Average Change %***		***-67.0***	***-74.6***	***-29.5***	***-35.8***	***-41.6***	***-0.1***	***-33.2***

Negative value indicates decrease and positive value indicates increase in trait values from Dalhart to Springlake.

The interaction between planting dates (normal *vs.* late) and clones in Springlake was non-significant for all the traits except specific gravity (**Supplementary Table 5**). Also, the traits measured did not vary with planting dates in Springlake, indicating that the two planting dates could not be distinguished based on the data measured and were equally stressful. Significant differences existed between clones for marketable yield, percent of tubers with external defects, average tuber weight, and specific gravity. Clones, planting dates, and their interaction explained around 13, 11, and 9% variation, respectively, in the total yield observed (**Supplementary Figure 3**).

**Figure 4:**
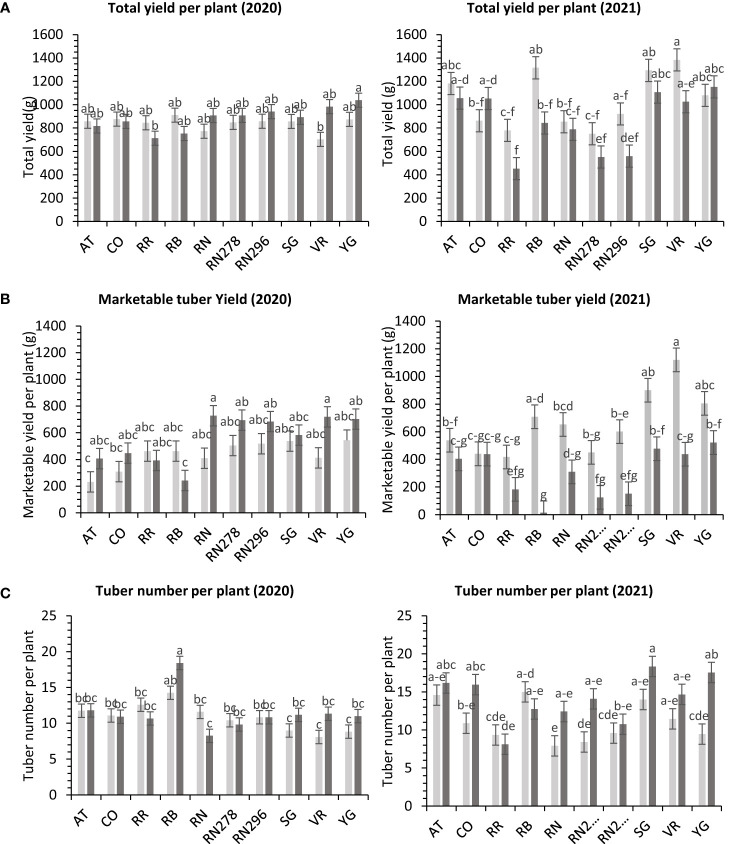
Least squares means of **(A)** tuber yield, **(B)** marketable yield and **(C)** tuber number per plant in ten potato clones (x-axis) grown at two different greenhouse conditions (Normal-light grey bars and heat stress - dark grey bars) in 2020 and 2021. Vertical bars represent mean ± SE. Bars with different letters differ significantly at p<0.05 (Tukey’s HSD).

The total yield of potatoes showed differential responses to temperature conditions (normal *vs.* heat stress) in greenhouses for the clones tested in both years. Clones, conditions, and their interactions explained variations in the trait, 5%, 1%, and 10%, respectively, in 2020; and 48%, 9%, and 10%, respectively, in 2021 (**Supplementary Figure 4**). The clones were significantly different for total yield in 2021. However, we failed to detect significant differences among clones for total yield in 2020. In 2020, Yukon Gold, under heated conditions, produced the highest weight of tubers per plant (1,038.4 g). There was no significant difference between clones for total yield per plant under normal conditions in the greenhouse in 2020 (**Figure 4**). In 2021, Vanguard Russet in normal conditions produced the highest weight of tubers per plant (1,383.4 g/plant), but Reveille Russet in heated conditions produced the lowest weight of tubers per plant (452.5 g/plant) (**Figure 4**).

#### 3.2.2 Marketable yield

Unlike total yield, there were significant differences between clones for marketable yield (US) when comparing locations throughout the years (**Supplementary Table 4**). Location explained most of the variation in marketable yield of the clones tested in different years compared with the genotype and their interaction (**Supplementary Figure 2**). Atlantic showed a consistently high marketable yield (note that internal issues such as internal heat necrosis were not detected based on an initial visual assessment of intact tubers). Vanguard Russet and several others produced statistically higher yields than Russet Burbank (zero marketable yield in 2019) in Dalhart (**Figure 3**). Dalhart had significantly higher total and marketable yields than Springlake (**Figure 3**).

**Figure 5:**
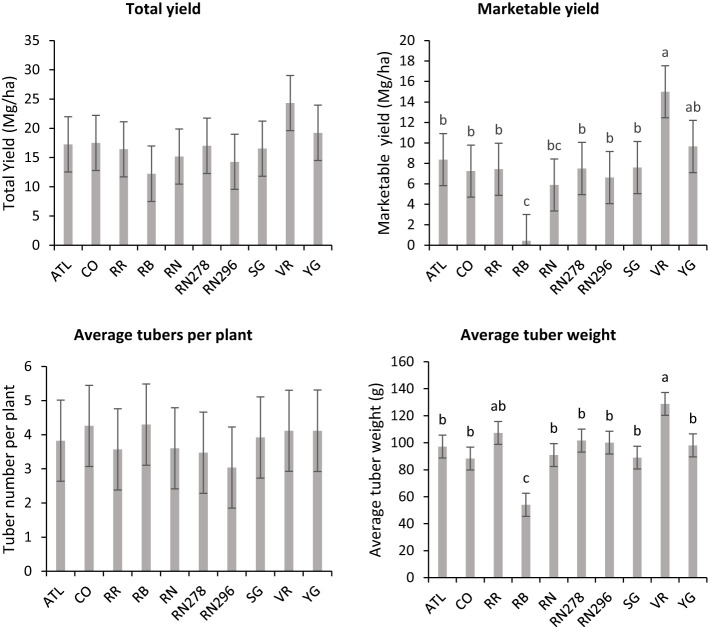
Least square means obtained from combined analysis on selected traits (total yield, marketable yield, average tubers per plant and average tuber weight) in ten potato clones grown at two different planting times (normal and late) in Springlake, TX, in 2019 and 2020. Vertical bars represent mean ± SE. Bars with different letters differ significantly at p<0.05 (Tukey’s HSD).

**Figure 6:**
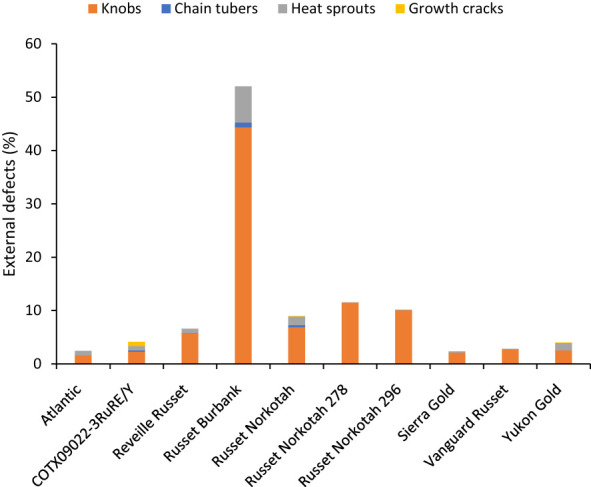
Distribution of the percentage of tubers with external defects by category based on assessments in Springlake, TX, with two planting dates (combined here since there were no significant differences for planting dates) in 2019 and 2020.

Unlike total yield, marketable yield varied with the clones tested (**Supplementary Table 5**) in regular and late planting trials in Springlake. Clone, planting dates, and their interaction explained around 26, 15, and 7% variation in the marketable yield (**Supplementary Figure 3**). The highest marketable yielding clone was Vanguard Russet (15 Mg/ha), whereas the lowest yielding clone was Russet Burbank (0.4 Mg/ha) (**Figure 5**). The percentage of tubers with external defects (PTED) varied among tested clones but did not vary significantly with planting dates (**Supplementary Table 5**). Clones alone explained around 55% of the variation for the percent tubers with external defects (**Figure 6**). The clone with the highest percentage of tuber defects was Russet Burbank (58.3%), and all other clones tested had statistically similar percentages of tubers with external defects (2.3% in Sierra Gold and 11.5% in Russet Norkotah 278) (**Figure 6**). The distribution of external tuber defects for each clone allowed us to quantify the most prevalent defects in each clone. Among the defects, knobs were the dominant form of external defects in all clones, followed by heat sprouts. Chain tubers and growth cracks were below 1% in all clones (**Figure 6**).

The total marketable yield of potatoes had a differential response to temperature conditions (normal *vs.* heat stress) for the clones tested in both years (**Supplementary Table 6**) in greenhouses. Clones, conditions, and their interactions explained about 27%, 8%, and 14%, variations in the trait respectively, for 2020 and 27%, 34%, and 11% respectively, for 2021 (**Supplementary Figure 4**). In 2020, Russet Norkotah (727.3 g/plant), along with Vanguard Russet (719.3 g/plant), in a heated condition, produced the highest weight of marketable tubers per plant. The lowest weight of tubers was produced by Russet Burbank (242.2 g/plant) in a heated condition, along with Atlantic (232.1 g/plant) in normal condition (**Figure 4**). In 2021, Vanguard Russet, in normal conditions, produced the highest weight of marketable tubers per plant (1,119.6 g/plant). In contrast, under heated conditions, Russet Burbank had the lowest weight of marketable tubers per plant (14.2 g/plant) (**Figure 4**).

#### 3.2.3 Percentage tubers with internal defects

**Figure 7:**
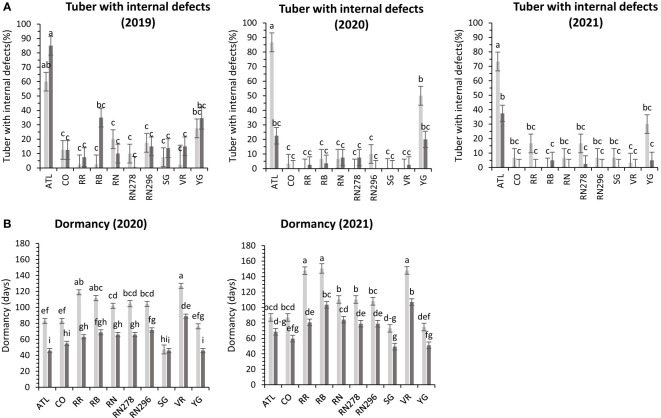
Least squares means of **(A)** percent tubers with internal defects, and **(B)** dormancy in ten potato clones (x-axis) grown at two different Texas locations (Dalhart - light grey bars and Springlake - dark grey bars) in 2019, 2020 and 2021. Vertical bars represent mean ± SE. Bars with different letters differ significantly at p<0.05 (Tukey’s HSD).

Internal defects included vascular discoloration, black spots, hollow heart, and internal heat necrosis. One or multiple defects were observed in tubers sampled for the defects. The data was recorded as the percentage of internal defects (PID) observed in ten tubers. Although location-by-clone interaction was significant in two out of three years’ trials, variation in internal defects was primarily attributed to the clone’s genetic makeup (**Supplementary Figure 2**). Dalhart gave a consistent value of around 16.4% internal tuber defects compared to more spread-out internal defects in Springlake (5 - 22.9%) (**Table 4**). Atlantic consistently had significantly higher internal defects in both locations during the years tested (**Figure 7**).

**Figure 8:**
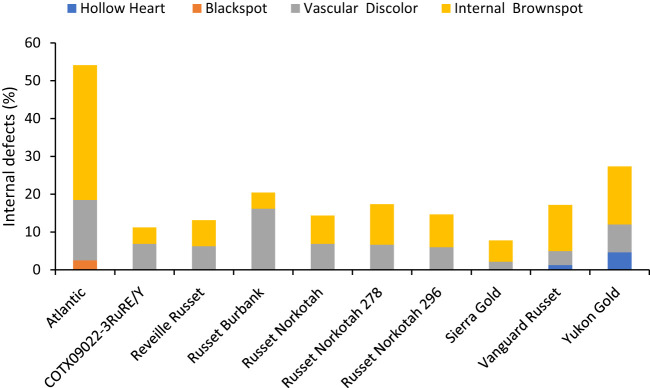
Distribution of the percentage of tubers with internal defects by category based on assessments in Springlake, TX, with two planting dates (combined here since there were no significant differences for planting dates) in 2019 and 2020.

The percentage of tubers with internal defects varied with clones tested (**Supplementary Table 5**). Clones, planting date, and their interaction explained around 33%, 4%, and 3% variation for the percent tubers with internal defects (**Supplementary Figure 3**). The distribution of tubers for each clone on the type of internal defects allowed us to quantify the most prevalent defects in each clone. The clone with the highest percentage of tuber defects was Atlantic (52.5%) and the lowest in Sierra Gold (7.8%). Internal brown spot or internal heat necrosis was most prevalent among the internal defects, followed by vascular discoloration. Blackspot was only observed in Atlantic at 2.5%; hollow heart was present in both Yukon gold (4.7%) and Vanguard Russet (1.3%) (**Figure 8**).

**Figure 9:**
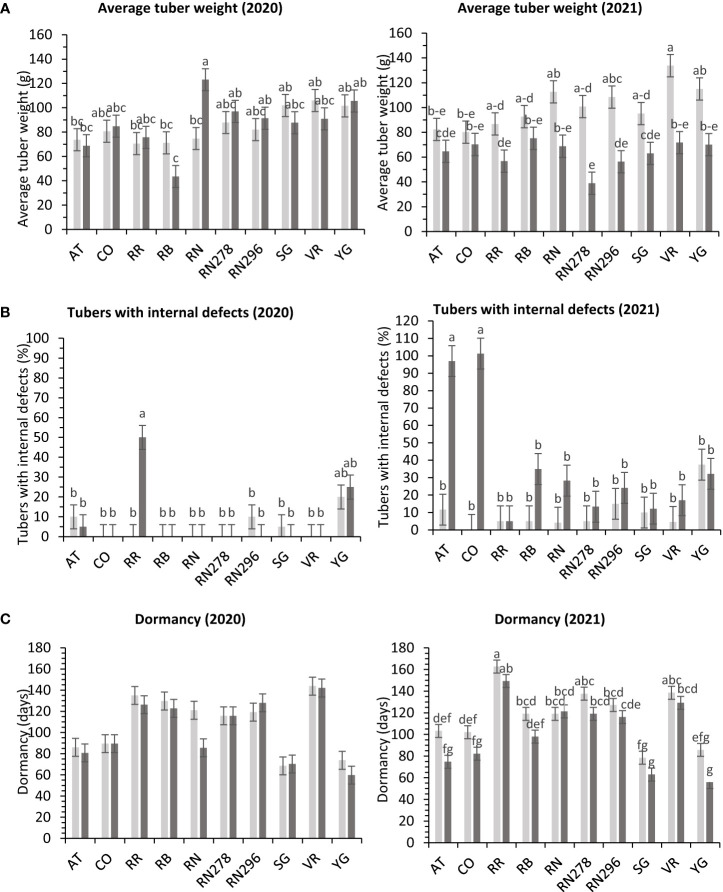
Least squares means of **(A)** external defects, **(B)** internal defects, and **(C)** Dormancy in ten potato clones (x-axis) grown at two different greenhouse conditions (Normal-light grey bars and heat stress- dark grey bars) in 2020 and 2021. Vertical bars represent mean ± SE. Bars with different letters differ significantly at p<0.05 (Tukey’s HSD).

In greenhouse trials, the percentage of tubers with internal defects had a differential response to temperature conditions (normal *vs.* heat stress) for the clones tested in both years (**Supplementary Table 6**). Clones, conditions, and their interactions explained variation in the trait by 31%, 1%, and 24%, respectively, for 2020 and 27%, 18%, and 31% for 2021 (**Supplementary Figure 4**). In 2020, Reveille Russet, under heated conditions, produced the highest percentage of tubers with internal defects (50%), along with Yukon Gold 25/20% (heated/normal conditions). All other clones had fewer tubers with internal defects (**Figure 9**). In 2021, COTX09022-3RuRE/Y (100%) and Atlantic (97%) produced the highest percentage of tubers with internal defects under heat stress. All other clones had a lower percent of tubers with internal defects, none in COTX09022-RuRE/Y under normal conditions and 37.5/32.2% (normal/heat stress) in Yukon Gold) (**Figure 9**).

#### 3.2.4 Average tuber number and tuber weight

**Figure 10:**
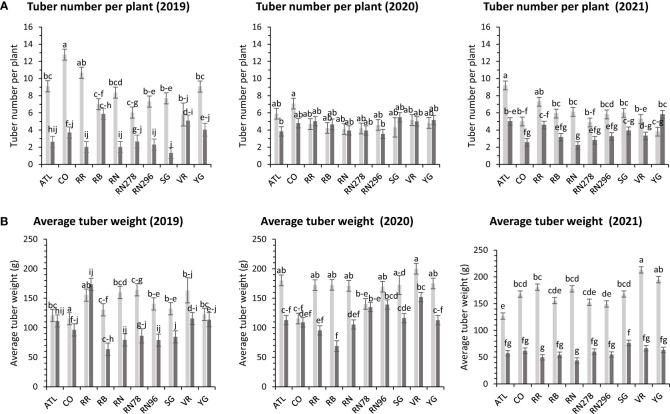
Least squares means of **(A)** tuber number per plant and **(B)** average tuber weight in ten potato clones (x-axis) grown at two different Texas locations (Dalhart - light grey bars and Springlake - dark grey bars) in 2019, 2020 and 2021. Vertical bars represent mean ± SE. Bars with different letters differ significantly at p<0.05 (Tukey’s HSD).

The average number of tubers per plant responded to variation in location, clone, and their interaction in two of the three years tested. In 2020, the average tuber number (ATN) per plant did not vary with location, clone, and their interaction. This inconsistency in explaining observed variation in average tuber number can be due to the greater variance of the experimental error in 2020, where the model failed to assign a significant source of variation to the factors involved in the experiment. However, for the other two years, location explained more than 60% of the variation in the trait (**Supplementary Figure 2**). In Dalhart, clones produced significantly more tubers (6.4) per plant than Springlake (3.8). Variation in average tuber weight was explained by location, clones, and their interaction in decreasing order each year of testing. Similar to the average tuber number per plant, the average tuber weight (ATW) was observed to be significantly higher in Dalhart (158.7 g) than in Springlake (91.4 g) (**Figure 10**).

There were no significant differences between clones for average tuber number per plant; however, there were significant differences between clones for average tuber weight (**Supplementary Table 5**) for trials related to (objective 2) assessing the effect of planting dates in Springlake. Clones produced a similar number of tubers, but the accumulation of photoassimilates in tubers varied per clone and is reflected in the average weight of tubers. Clone, planting date, and their interaction explained around 31%, 15%, and 5% variation for the percent tubers with average tuber weight (**Supplementary Figure 3**). Vanguard Russet (129 g) and Reveille Russet (107 g) were the highest average tuber weight clones. Russet Burbank, however, had the lowest average tuber weight (54 g) (**Figure 5**).

In greenhouse trials (objective 3), the average tuber number per plant and average tuber weight displayed varied responses to temperature conditions across tested clones in both years (**Supplementary Table 6**). For 2020, clones, conditions, and their interactions accounted for approximately 44%, 1%, and 16% of the variation in average tuber number. Similarly, in 2021, these factors explained about 34%, 15%, and 15% of the variation (**Supplementary Figure 4**). In 2020, Russet Burbank exhibited the highest tuber number per plant (18.4) under heated conditions, while Sierra Gold had the highest (18.3) in 2021, also under heated conditions. Russet Norkotah showed the lowest tuber number per plant in 2020 (8.3) under heat stress and in 2021 (7.9) under normal conditions (**Figure 4**). Regarding average tuber weight, in 2020, clones, conditions, and their interactions accounted for 32%, 1%, and 15% of the variation, respectively. In 2021, these factors explained 12%, 42%, and 10% of the variation (**Supplementary Figure 4**). In 2020, Russet Norkotah had the highest average tuber weight (123.1 g) under heat stress, while Vanguard Russet recorded the highest (133.8 g) under normal conditions in 2021. Conversely, Russet Burbank (43.6 g) in 2020 and Russet Norkotah 278 (39 g) in 2021 under heat stress had the lowest average tuber weight (**Figure 9**).

#### 3.2.5 Specific gravity and dry matter

Variation in specific gravity was assigned to genetics more than location and clone*location interaction. About 50% of the variation in specific gravity was governed by the genetics of the tested clones, whereas between 11 to 17% of the variation was due to the interaction of location and clone and the location itself (**Supplementary Figure 2**). Atlantic had consistently higher specific gravity, whereas clones like Russet Norkotah and Vanguard Russet had the lowest specific gravity.

Specific gravity exhibited differential responses of clones to planting times (regular *vs.* late). Atlantic under late planting had the highest specific gravity (1.076) compared to the lowest specific gravity in Russet Burbank (1.051) under regular planting. Clones explained around 26% variation in specific gravity; however, planting dates and planting dates*clone explained only 13% and 11%, respectively.

Results from greenhouse trials for studying the effects of normal *vs.* heat stress conditions on specific gravity and tuber dry matter of potato clones have been previously reported (Gautam et al., 2023). Results showed that clones and growing conditions were major factors affecting the phenotypic variation for both traits (**Supplementary Figure 4**), with heat stress conditions resulting in lower specific gravity and tuber dry matter than normal conditions. Some clones had differential responses to the growing conditions. Atlantic had the highest specific gravity and tuber dry matter, while Russet Burbank was the most affected by the reduction in both traits under heat stress. The tuber dry matter was measured only in the controlled greenhouse trials (normal *vs.* heat stress) and was used to obtain the harvest index for the greenhouse trial. Since tuber dry matter is highly correlated with specific gravity, tubers produced under heat stress conditions had significantly lower tuber dry matter than those harvested from normal growing conditions, similar to the results on specific gravity.

#### 3.2.6 Dormancy

The dormancy period length (DOR) of the tubers harvested from field trials and stored at room temperature was evaluated in 2020 and 2021. Variations in dormancy were observed among potato clones grown at two locations in Texas (**Supplementary Table 4**). Location, clones, and their interactions explained significant proportions of dormancy variation in both years, with location accounting for more than one-third of the variation. Dalhart-grown potatoes exhibited longer (103 days) dormancy periods compared to those grown in Springlake (69 days), with an average reduction of 33% between the two locations. Vanguard Russet tubers harvested in Dalhart had the longest dormancy periods in both years, while Sierra Gold, Yukon Gold, Atlantic, and COTX09022-3RuRE/Y in Springlake had the shortest dormancy periods (**Figure 7**).

Evaluation of the length of the dormant period (based on tubers stored at room temperature) of tubers harvested from planting date trials in Springlake (objective 2) was evaluated only in 2020. Clones, planting date, and their interaction explained around 79%, 2%, and 11%, respectively, variation for the dormancy (**Supplementary Figure 3**). In both the planting dates, Vanguard Russet had the most extended dormancy: 85 and 88 days, respectively, for normal and late planting, together with Reveille Russet (89 days). Atlantic and Yukon Gold had the shortest dormancy (38 days) under late planting (**Supplementary Figure 5**).

Significant differences were observed in the length of tuber dormancy periods between years (objective 3) (**Supplementary Table 6**) in tubers harvested from the greenhouse. In 2020, clones accounted for approximately 70% of the variation in the dormancy period, with conditions and clone-condition interactions explaining only 1% and 4% of the variation, respectively (**Supplementary Figure 4**). Yukon Gold (67 days), Sierra Gold (69 days), and Atlantic (83 days) exhibited the shortest dormancy periods, while Vanguard Russet had the longest (144 days). In 2021, clones primarily explained dormancy variation (77%), with conditions and clone-condition interactions contributing 7% and 2%, respectively. Normal conditions (117 days) favored longer dormancy than heated conditions (101 days). Yukon Gold (71 days), Sierra Gold (71 days), and Atlantic (89 days) again had the shortest dormancy periods. In comparison, Reveille Russet exhibited the longest (156 days), followed by Vanguard Russet (134 days) and Russet Norkotah 278 (128 days) (**Figure 9**).

#### 3.2.7 GGE-biplots and multi-trait stability index

The GGE-Biplots method was used to visualize the relationship between environments and clone performance for measured traits (**Supplementary Figures 5, 6**), explaining variation from 63.01% in Average tuber weight (ATW) to 98.54% in the percentage of cull tubers (PCU). The plots revealed similarities between Springlake and Dalhart in most traits, except for average tuber number and weight. Springlake environments appeared more discriminating for marketable and cull tuber percentages, while Dalhart showed higher total yields but more internal defects. Vanguard Russet performed well in most environments, while Russet Burbank and Atlantic performed poorly due to high non-marketable tuber percentages (Russet Burbank) and internal defects (Atlantic). COTX09022-3RuRE/Y consistently produced the most tubers per plant.

**Figure 11:**
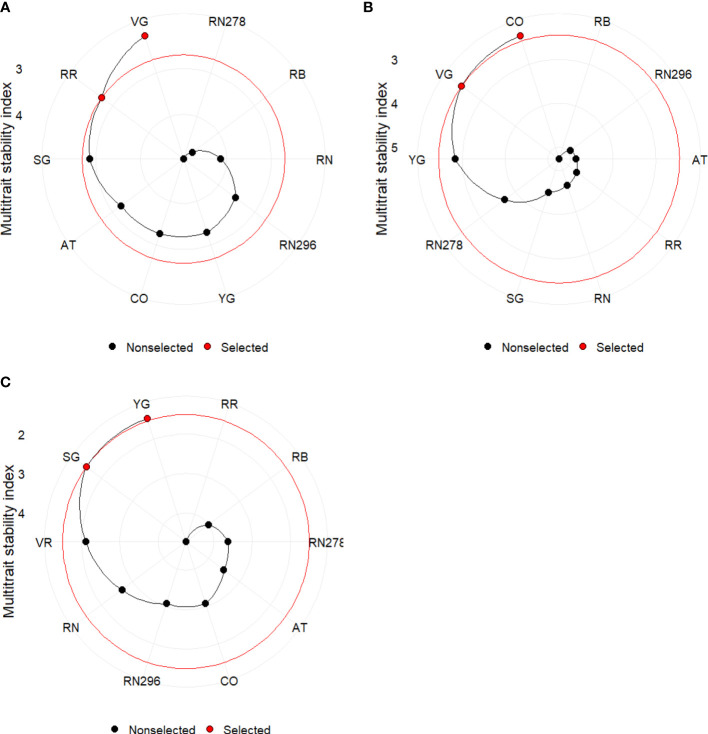
Genotype ranking and selected genotypes for the multi-trait stability index considering a selection index of 20% for potatoes grown **(A)** in two locations (Springlake *vs.* Dalhart) during 2019, 2020 and 2021; **(B)** at two planting times (regular *vs.* late) in Springlake during 2019 and 2020; **(C)** under greenhouse conditions (normal *vs.* heat stress) during 2020 and 2021. AT, Atlantic; CO, COTX09022-3RuRE/Y; RR, Reveille Russet; RB, Russet Burbank; RN, Russet Norkotah; RN278, Russet Norkotah 278; RN296, Russet Norkotah 296; SG, Sierra Gold; VR, Vanguard Russet; YG, Yukon Gold.

The multi-trait stability index (MTSI) for multi-environment trials (using a mixed model based on mean performance and stability) allowed a robust selection of genotypes. The index was built using yield traits (TY, US, CU, PUS, PCU, ATN, ATW) and quality (PTED, PID), including specific gravity (SG). Positive traits were TY, US, PUS, ATN, ATW and SG, while negative traits were CU, PCU, PTED, and PID during the calculation of MTSI. The selection percentage was set to 20%. Based on MTSI, the clones selected based on locations were Vanguard Russet and Reveille Russet, while those based on planting dates were COTX09022-3RuRE/Y and Vanguard Russet. For the greenhouse trials, the selected clones were Yukon Gold and Sierra Gold, followed by Vanguard Russet (**Figure 11**).

## 4 Discussion

Our research subjected potatoes to prolonged heat stress in field and greenhouse experiments, aligning with studies by Tang et al., 2018 and Bashir et al., 2023. Unlike many short-term heat stress treatments, we employed extended periods throughout the crop cycle, consistent with findings that temperatures above 25°C stress potatoes. Temperature regimes used by Boguszewska-Mańkowska et al., 2020; Zhang et al., 2021, and Rykaczewska, 2015 reflect similar stress conditions. Some of the field sites that reported heat stress on potatoes are San Ramón, Peru, and Lavras, Brazil. San Ramón frequently experiences heat stress (Khan et al., 2015; Benavides et al., 2017; Raymundo et al., 2018; Muñoa et al., 2022) with an average day/night temperature of 28/23°C (Benavides et al., 2017) but fluctuating between 16 to 36°C (Muñoa et al., 2022). In Lavras, Brazil, the mean maximum and minimum temperatures are 28.5 and 16.5°C, respectively (Lambert et al., 2006). Both the locations in our study encounter prolonged periods of temperature exceeding 25°C throughout the crop period and often reaching above 40°C (Gautam et al., 2024). High temperatures inhibit potato tuber growth and development in short- and long-day conditions (Wheeler and Tibbitts, 1986), with the inhibitory effect more significant in long-day conditions (Van Dam et al., 1996; Jackson, 1999) as in our study. Although there are reports of episodes of heat stress during germination, tuber initiation, and bulking or maturation, few sites observe a constant increase in temperature from planting to harvest, as in our case. Springlake exhibits a steady rise in temperature and photoperiod throughout the crop period. While Dalhart also faces high temperatures, the photoperiod consistently decreases from tuberization to harvest, mitigating some adverse effects of heat on tuber development. Springlake’s combination of high temperature and increased photoperiod during crop growth positions it uniquely. This scenario may become more prevalent in the future due to climate change in the northern hemisphere potato-producing region. Therefore, the field sites we utilized could serve as representative of future temperature scenarios for most potato-growing regions in the Northern hemisphere. The GGE biplot on the relationship of environments (type 10) also reveals that Springlake environments are more consistent and can discriminate clones, primarily based on tuber defect traits (**Supplementary Figure 6**), thus making it suitable for future heat stress-related studies.

Environment, cultivar, and their interaction jointly dictate yield capacity (Benavides-Cardona et al., 2022), with environment notably impacting traits like yield and tuber dry matter. A yield reduction of 13% in Brazil (Patino-Torres et al., 2021) was recorded, while in Bangladesh, reductions ranged from 4.5% to 34.8% (Mahmud et al., 2021) due to heat stress. The yield in the current study was higher in field and greenhouse experiments exposed to less heat stress, consistent with findings from previous reports. Our study, conducted under more stressful conditions, observed a substantial 67% (43% to 84%) yield reduction between Dalhart and Springlake, with no significant reduction observed with different planting times at Springlake. Greenhouse conditions in 2021 led to an 18% yield reduction, and a similar study reported a 24% reduction under comparable conditions (Rykaczewska, 2015). Potato clones had significantly higher total yields in Dalhart than in Springlake. Lower temperatures within the 12-28°C favorable range correspond to higher potato yields, indicating an increased harvest index (Wheeler and Tibbitts, 1986). Our greenhouse experiments showed that heat stress reduced the harvest index of potatoes by ~8% in 2020 and 20.5% in 2021 (**Supplementary Table 7**). The conditions in 2021 were more contrasting than in 2020 in the greenhouses. Thus, a greater harvest index reduction (from normal to heat stress) was realized in 2021. Under high-temperature conditions, the potato plants had greater aerial mass, resulting in a lower harvest index. The above-ground dry mass increased by 53% in 2020 and 67% in 2021. This increase in above-ground dry mass could be explained by the increased plant height (~33% in 2020 and 30% in 2021) and a decrease in leaf area by 73% in 2021 (**Supplementary Table 7**). Photoassimilates were diverted to aerial rather than underground parts (tubers) under heat-stress conditions. Several reports (Wolf et al., 1990; Hancock et al., 2014; Aien et al., 2017) corroborate this.

There are reports of tuber quality deterioration in potatoes due to heat stress. High temperatures inhibit tuber formation, deform tubers, and cause chain tuber formation, secondary growth, and premature sprouting (Bodlaender, 1963; Levy and Veilleux, 2007; Rykaczewska, 2015; Zhang et al., 2021). Tuber deformities include knobby tubers, heat sprouts, chained tubers, and growth cracks. Knobby tubers result from interrupted growth, resulting in shape deformation with irregularly shaped secondary growth or bumps, while heat sprouts occur when tubers sprout prematurely due to heat exposure. Chained tubers form when tuber growth is periodically interrupted, and growth cracks emerge when the tuber splits and heals (Hutchinson, 1969; Van Den Berg et al., 1990). Heat stress conditions can lead to tuber deformities, with reported percentages varying from 0% to 40% (Rykaczewska, 2015) and 9.6% to 16.1% (Patino-Torres et al., 2021). Genotypes vary in their capacity to tolerate heat, and sensitive varieties can suffer a substantial marketable yield loss. This study presents the first comprehensive quantification of various tuber defects in potatoes under prolonged field and greenhouse heat stress. Tuber defects prevalent under heat-stress field conditions were similarly observed in heat-stress greenhouse conditions, highlighting the correlation between high temperatures and tuber defects. The dissection of defects into categories like heat sprouts, chain tubers, knobs, and growth cracks, along with their distribution with non-marketable tubers, is novel. While Dalhart had consistent internal tuber defect percentages (~16%), Springlake exhibited variability (5-23%) across years, suggesting multifactorial influences on defect expression. Despite Atlantic’s high total and marketable yields, it exhibited a high percentage of internal defects, particularly internal heat necrosis, indicating susceptibility to high temperatures.

Heat stress in potatoes can decrease yield due to multiple factors. Elevated temperatures during tuber formation decreased the number of tubers per plant (Zhang et al., 2021), and in extreme cases, tuber initiation is inhibited. Additionally, heat stress may delay tuber initiation, resulting in the formation of numerous smaller tubers instead of larger ones (Mahmud et al., 2021), as we found in our experiments. Dalhart produced a higher number of large tubers than Springlake. The conditions in Dalhart favored better initiation of tuberization and bulking than Springlake. Late planting in Springlake reduced tuber weight, indicating reduced assimilate allocation to tubers, although the number of tubers remained unaffected. The greenhouse trials corroborate the results from the field. Less extreme conditions in 2020 did not show significant differences in tuber number and weight, but more contrasting conditions in 2021 showed significant differences. Lafta and Lorenzen (1995) observed that high temperatures caused fewer tubers to be formed. However, some studies, like that of Borah and Milthorpe (1962) and Struik et al. (1989), report that more tubers are formed at higher temperatures (Struik, 2007). There was more than double (99% to 143%) increase in tuber numbers, with individual tuber mass ranging from 58% to 98% of the control when six cultivars of potatoes were grown under heat stress for 14 days (Rykaczewska, 2015). At first glance, the results seem contradictory: Heat stress could reduce the average number of tubers per plant or increase the number per plant. The severity and timing of heat stress and genotypic differences for time to tuber initiation could explain the results. In some clones, high temperatures around the developmental stage of tuber initiation could inhibit tuber formation or severely reduce the number of tubers formed. However, clones with very early initiation of tuberization could escape heat stress. On the other hand, clones with later tuber initiation could end up with a higher number of smaller tubers.

Increased temperatures affect the dry matter distribution in potatoes, favoring above-ground growth over underground tubers (Marinus and Bodlaender, 1975). It is assimilate partitioning, not the total plant productivity, that is affected by high temperature, as confirmed by an increase in translocation of radiolabeled C to vegetative organs (leaves and stems) along with a decrease towards the tubers (Wolf et al., 1990). We confirmed this discriminatory partitioning of assimilates through the harvest index. The reduced harvest index under heat stress confirms assimilates diversion to aerial parts over economic tubers. An indirect method of estimating tuber dry matter can be done by measuring the specific gravity of tubers (Nzaramba et al., 2013). Our specific gravity measurements in field and greenhouse settings and tuber dry matter assessments in greenhouse trials show a decrease due to heat stress. Higher specific gravity is desired in potatoes, especially in processing types, as it indicates higher dry matter in the tuber and less oil absorption in fried/processed products. The specific gravity of potatoes decreases with high-temperature stress (Teixeira et al., 2015; Andrade et al., 2021; Fernandes Filho et al., 2021). Various studies indicate a drastic reduction in tuber dry matter when potatoes are grown above 20-25°C (Levy and Veilleux, 2007; Aien et al., 2017; Kim and Lee, 2019; Obiero et al., 2019). Dalhart was more favorable than Springlake regarding specific gravity. Planting dates did not have significant differences in the specific gravity of the samples tested in Springlake, indicating similar heat stress conditions during both planting dates. However, the clones showed significant differences in specific gravity in the field and greenhouse trials. The majority of variation explained by clones in all three trials indicate genetic control of the trait. Thus, observing more genetic control of specific gravity would allow breeders to develop varieties with high specific gravity and, therefore, high dry matter. The synthesis of high levels of endogenous gibberellins, which reduces the partitioning of assimilates to the tubers and impedes the synthesis of starch and tuber-specific proteins, has been associated with the reduction of tuber dry matter under high temperatures (Lovell and Booth, 1967).

Potato dormancy is regulated by a combination of genetic factors, environment, physiology, and hormones (Sonnewald and Sonnewald, 2014). The current study showed that dormancy in potatoes is mainly controlled genetically. However, location and growth conditions (planting dates and temperatures) influenced tuber dormancy. High temperatures during tuber growth in the field lead to reduced dormancy, indicating physiological aging (Midmore and Roca, 1992; Van Ittersum et al., 1992; Suttle, 2007). In a study, varieties Premier Russet and Ranger Russet, when exposed to high temperatures (29°C), shortened their dormancy compared to normal (16°C) temperature during the tuber maturity stage (Zommick et al., 2014). In 2020, with less extreme temperatures, the effects on dormancy in potato clones were not fully realized. However, the subsequent 2021 trial revealed that extreme temperatures significantly shortened dormancy in the potato clones. On its extreme effect, high temperature can break dormancy in tubers still growing in the field, often termed “heat sprouts” (Levy and Veilleux, 2007; Zhang et al., 2021). Greenhouse experiments and field trials showed consistent results. In both settings, more extreme conditions (Springlake, late planting and heated conditions in 2021) led to shorter dormancy in potato clones than milder conditions (Dalhart, regular planting and normal conditions in 2021). The shortening of dormancy and heat sprouting in the field due to heat stress is likely associated with the gibberellin biosynthesis pathway (Zhang et al., 2021). Many studies consider postharvest temperatures critical for prolonging tuber dormancy in potatoes; however, the importance of temperatures during growth can be highlighted through studies like ours.

Our results were consistent with findings that the environment explains a significant part of the variation for traits like total yields, marketable yield, and dry matter, while genotypes contribute substantially (Sood et al., 2020). Based on the range of observed characteristics, we propose three levels of potato heat tolerance. The first level pertains to a clone’s ability to yield, the second level involves producing high yield without external deformities, and the third level encompasses the production of tubers free from both external and internal defects. Russet Burbank consistently displayed poor marketable yield, whereas Vanguard Russet emerged as a top-yielding variety with more marketable tubers across various environments. Reveille Russet exhibits unique traits such as long dormancy and late emergence. Its late emergence may mask its potential heat tolerance (Gautam et al., 2021). Early exposure to high temperatures and premature tuber harvesting can reduce tuber marketable yield, specific gravity, dry matter, and dormancy. Despite successful evaluation in lab conditions (Gautam et al., 2021), assessing Reveille Russet for heat tolerance in the field and greenhouse remains challenging due to its shorter growth period. In greenhouse trials, Sierra Gold and Yukon Gold were identified as high-yielding clones using a multi-trait stability index, with shorter maturation periods than other russet varieties. Russet Burbank’s sensitivity to heat stress in Texas growing sites is consistent with its optimal temperature of 17.5°C (Yandell et al., 1988) for yield. Increased heat sprouting in later stages of crop maturity (Rykaczewska, 2015) suggests its correlation with prolonged heat stress, explaining its lesser detection in greenhouse trials due to shorter growth periods (90 days) compared to 100 or more days in the field. Considering various measured traits, Vanguard Russet stands out for its high marketable tuber yield, average tuber weight, and long dormancy with few internal defects. Conversely, Russet Burbank exhibited low marketable tuber yield and many small tubers, along with reduced specific gravity under heat stress. Although Atlantic produced a high yield of external defect-free tubers, it was sensitive to internal defects, indicating heat susceptibility. These results are effectively summarized using GGEbiplot type 3: Which won Where plot (**Supplementary Figure 7**) and multi-trait stability index plots (**Figure 11**), confirming the consistent performance of Vanguard Russet across field and greenhouse experiments.

## 5 Conclusions

Potato plants grown in the Panhandle region of Texas (represented by the Springlake and Dalhart locations) face temperatures exceeding 25°C during the growing season. Both locations are suitable for heat stress screening. Springlake stands out as the most stressful site for heat stress evaluation, and regular planting time is suitable for heat stress screening. Greenhouse trials confirmed field findings on the effect of heat stress on tuber yield, quality, and dormancy. Vanguard Russet is a reliable heat-tolerant reference. Russet Burbank and Atlantic can be used as sensitive checks due to their high incidence of external (Russet Burbank) and internal (Atlantic) defects. Heat stress during the potato growing season significantly reduces marketable yield, increases external and internal tuber defects, alters the chemical composition (lower specific gravity, dry matter, starch, higher percent of simple sugars), and reduces dormancy. Heat-tolerant cultivars are less affected by the detrimental effects of heat stress.

Some limitations of this study include the difficulty of achieving normal/ideal potato growing conditions (25/15°C) under greenhouse conditions. The normal/ideal conditions were moderately stressful. A possible confounding effect was the inclusion of genotypes from different plant maturity groups and market classes. Under greenhouse conditions, all clones were harvested at 90 DAP, and some clones did not achieve full potential (i.e. Reveille Russet). Despite the study’s limitations, key messages on the effect of heat stress in potatoes can be drawn, and they are useful for further investigations on screening genotypes for heat tolerance and understanding the underlying mechanism of heat tolerance in potatoes using potato clones contrasting for heat tolerance.

## Data availability statement

The raw data supporting the conclusions of this article will be made available by the authors, without undue reservation.

## Author contributions

SG: Conceptualization, Data curation, Formal analysis, Investigation, Methodology, Visualization, Writing – original draft, Writing – review & editing. DS: Data curation, Formal analysis, Investigation, Methodology, Project administration, Writing – original draft, Writing – review & editing. JK: Data curation, Formal analysis, Investigation, Methodology, Project administration, Writing – original draft, Writing – review & editing. MV: Conceptualization, Funding acquisition, Investigation, Methodology, Project administration, Resources, Supervision, Visualization, Writing – original draft, Writing – review & editing.

## References

[B1] Ahn Y. J. Claussen K. Lynn Zimmerman J. (2003). Genotypic differences in the heat-shock response and thermotolerance in four potato cultivars. Plant Sci. 166, 901–911. doi: 10.1016/j.plantsci.2003.11.027

[B2] Aien A. Chaturvedi A. K. Bahuguna R. N. Pal M. (2017). Phenological sensitivity to high temperature stress determines dry matter partitioning and yield in potato. Indian J. Plant Physiol. 22, 63–69. doi: 10.1007/s40502-016-0270-z

[B3] Andrade M. H. M. L. Patino-Torres A. J. Cavallin I. C. Guedes M. L. Carvalho R. P. Gonçalves F. M. A.. (2021). Stability of potato clones resistant to potato virus Y under subtropical conditions. Crop Breed. Appl. Biotechnol. 21, 1–9. doi: 10.1590/1984-70332021v21n1a8

[B4] Bashir I. Nardino M. Castro C. M. Heiden G. (2023). Genotypic response and selection of potato germplasm under heat stress. Potato Res. 66, 85–104. doi: 10.1007/s11540-022-09573-w

[B5] Battisti D. S. Naylor R. L. (2009). Historical warnings of future food insecurity with unprecedented seasonal heat. Science 80)323, 240–244. doi: 10.1126/science.1164363 19131626

[B6] Benavides-Cardona C. A. Marcillo-Paguay C. A. Gómez-Gil L. F. Romero J. V. (2022). Physiological and yield response to fertilization of short-cycle *Solanum tuberosum* cultivars in three high-Andean environments. Rev. Fac. Nac. Agron. Medellin 75, 10009–10021. doi: 10.15446/rfnam.v75n3.99191

[B7] Benavides M. A. G. Diaz L. Burgos G. Felde T. Zum Bonierbale M. (2017). Heritability for yield and glycoalkaloid content in potato breeding under warm environments. Open Agric. 2, 561–570. doi: 10.1515/opag-2017-0059

[B8] Bhardwaj V. Rawat S. Tiwari J. Sood S. Dua V. K. Singh B.. (2022). Characterizing the potato growing regions in India using meteorological parameters. Life 12, 1–11. doi: 10.3390/life12101619 PMC9605082 36295054

[B9] Bodlaender K. B. A. (1963). Influence of temperature, radiation and photoperiod on development and yield. Growth Potato, 199–210.

[B10] Boguszewska-Mańkowska D. Gietler M. Nykiel M. (2020). Comparative proteomic analysis of drought and high temperature response in roots of two potato cultivars. Plant Growth Regul. 92, 345–363. doi: 10.1007/s10725-020-00643-y

[B500] Borah M. N. Milthorpe F. L. (1962). Growth of the potato as influenced by temperature. Indian J. Plant Physiol. 5, 53–72.

[B11] Brown C. R. (2015). Russet burbank: no ordinary potato. HortScience 50, 157–160. doi: 10.21273/HORTSCI.50.2.157

[B12] Bushnell J. (1925). The relation of temperature to growth and respiration in the potato plant. Am. Potato J. 4, 119–119. doi: 10.1007/BF02910567

[B13] Devaux A. Goffart J. P. Kromann P. Andrade-Piedra J. Polar V. Hareau G. (2021). The potato of the future: Opportunities and challenges in sustainable agri-food systems. Potato Res. 64, 681–720. doi: 10.1007/s11540-021-09501-4 34334803 PMC8302968

[B14] Dutt S. Manjul A. S. Raigond P. Singh B. Siddappa S. Bhardwaj V.. (2017). Key players associated with tuberization in potato: potential candidates for genetic engineering. Crit. Rev. Biotechnol. 37, 942–957. doi: 10.1080/07388551.2016.1274876 28095718

[B15] FAO (2019)FAO global statistical yearbook. In: FAO regional statistical Yearbooks. *FAOSTAT* . Available online at: http://www.fao.org/faostat/en/#data/QC (Accessed May 4, 2021).

[B16] Fernandes Filho C. C. Andrade M. H. M. L. Souza Marçal T. Fernandes M. O. Bastos A. J. R. Guedes M. L.. (2021). Selection of potato clones for heat tolerance and resistance to potato viruses X and Y for processing purposes. Crop Sci. 61, 552–565. doi: 10.1002/csc2.20361

[B17] Fumia N. Pironon S. Rubinoff D. Khoury C. K. Gore M. A. (2022). and Kantar, M Wild relatives of potato may bolster its adaptation to new niches under future climate scenarios. B. Food Energy Secur. 11, 1–15. doi: 10.1002/fes3.360

[B18] Gautam S. Morey R. Rau N. Scheuring D. C. Kurouski D. Vales M. I. (2023). Raman spectroscopy detects chemical differences between potato tubers produced under normal and heat stress growing conditions. Front. Plant Sci. 14. doi: 10.3389/fpls.2023.1105603 PMC9995913 36909401

[B19] Gautam S. Pandey J. Scheuring D. C. Koym J. W. Vales M. I. (2024). Genetic basis of potato tuber defects and identification of heat-tolerant clones. Plants 13, 616. doi: 10.3390/plants13050616 38475462 PMC10934851

[B20] Gautam S. Solis-Gracia N. Teale M. K. Mandadi K. Silva J. A. Vales M. I. (2021). Development of an *in vitro* microtuberization and temporary immersion bioreactor system to evaluate heat stress tolerance in potatoes (*Solanum tuberosum* L.). Front. Plant Sci. 12. doi: 10.3389/fpls.2021.700328 PMC8385365 34456944

[B21] Ghosh S. C. Asanuma K. Kusutani A. Toyota M. (2000). Effects of temperature at different growth stages on nonstructural carbohydrate, nitrate reductase activity and yield of potato. Environ. Control Biol. 38, 197–206. doi: 10.2525/ecb1963.38.197

[B22] Hancock R. D. Morris W. L. Ducreux L. J. M. M. Morris J. A. Usman M. Verrall S. R.. (2014). Physiological, biochemical and molecular responses of the potato (*Solanum tuberosum*L.) plant to moderately elevated temperature. Plant Cell Environ. 37, 439–450. doi: 10.1111/pce.12168 23889235

[B23] Hijmans R. J. (2003). The effect of climate change on global potato production. Am. J. Potato Res. 80, 271–279. doi: 10.1007/BF02855363

[B24] Hutchinson C. M. (1969). Potato physiological disorders - growth cracks. EDIS 2003, 1–2. doi: 10.32473/edis-hs182-2003

[B25] Jackson S. D. (1999). Multiple signaling pathways control tuber induction in potato. Plant Physiol. 119, 1–8. doi: 10.1104/pp.119.1.1 9880339 PMC1539201

[B26] Jennings S. A. Koehler A. K. Nicklin K. J. Deva C. Sait S. M. (2020). and Challinor, A Global potato yields increase under climate change with adaptation and CO2 fertilization. J. Front. Sustain. Food Syst. 4. doi: 10.3389/fsufs.2020.519324

[B27] Johansen R. H. Farnsworth B. Nelson D. C. Secor G. A. Gudmestad N. (1988). and Orr, P Russet Norkotah: A new russet-skinned potato cultivar with wide adaptation. H. Am. Potato J. 65, 597–604. doi: 10.1007/BF02908344

[B28] Johnston G. R. Rowberry R. G. (1981). Yukon Gold: A new yellow-fleshed, medium-early, high quality table and French-fry cultivar. Am. Potato J. 58, 241–244. doi: 10.1007/BF02853905

[B29] Khan M. A. Munive S. Bonierbale M. (2015). Early generation *in vitro* assay to identify potato populations and clones tolerant to heat. Plant Cell. Tissue Organ Cult. 121, 45–52. doi: 10.1007/s11240-014-0677-z

[B30] Kim Y. U. Lee B.-W. (2019). Differential mechanisms of potato yield loss induced by high day and night temperatures during tuber initiation and bulking: photosynthesis and tuber growth. Front. Plant Sci. 10. doi: 10.3389/fpls.2019.00300 PMC6426786 30923532

[B31] Kim Y. U. Seo B. S. Choi D. H. Ban H. Y. Lee B. W. (2017). Impact of high temperatures on the marketable tuber yield and related traits of potato. Eur. J. Agron. 89, 46–52. doi: 10.1016/j.eja.2017.06.005

[B32] Kroeger C. (2023). Heat is associated with short-term increases in household food insecurity in 150 countries and this is mediated by income. Nat. Hum. Behav. 7, 1777–1786. doi: 10.1038/s41562-023-01684-9 37604991 PMC10593604

[B33] Lafta A. M. Lorenzen J. H. (1995). Effect of high temperature on plant growth and carbohydrate metabolism in potato. Plant Physiol. 109, 637–643. doi: 10.1104/pp.109.2.637 12228617 PMC157630

[B34] Lambert E. D. S. Pinto C. A. B. P. De Menezes C. B. (2006). Potato improvement for tropical conditions: I. Analysis of stability. Crop Breed. Appl. Biotechnol. 6, 129–135. doi: 10.12702/1984-7033

[B35] Lehretz G. G. Sonnewald S. Lugassi N. Granot D. Sonnewald U. (2021). Future-proofing potato for drought and heat tolerance by overexpression of Hexokinase and SP6A. Front. Plant Sci. 11. doi: 10.3389/fpls.2020.614534 PMC7835534 33510758

[B36] Levy D. (1986). Genotypic variation in the response of potatoes (*Solanum tuberosum* L.) to high ambient temperatures and water deficit. F. Crop Res. 15, 85–96. doi: 10.1016/0378-4290(86)90103-6

[B37] Levy D. Veilleux R. E. (2007). Adaptation of potato to high temperatures and salinity-a review. Am. J. Potato Res. 84, 487–506. doi: 10.1007/BF02987885

[B38] Lobell D. B. Field C. B. (2007). Global scale climate–crop yield relationships and the impacts of recent warming. Environ. Res. Lett. 2, 14002. doi: 10.1088/1748-9326/2/1/014002

[B39] Lovell P. H. Booth A. (1967). Effects of Gibberellic acid on growth, tuber formation and carbohydrate distribution in *Solanum tuberosum* . New Phytol. 66, 525–537. doi: 10.1111/j.1469-8137.1967.tb05424.x

[B40] Mahmud A. Jahangir Alam M. Kundu B. C. Skalicky M. Matiar Rahman M. Shofiur Rahaman E. H. M.. (2021). Selection of suitable potato genotypes for late-sown heat stress conditions based on field performance and stress tolerance indices. Sustain. 13, 1–14. doi: 10.3390/su13052770

[B41] Marinus J. Bodlaender K. B. A. (1975). Response of some potato varieties to temperature. Potato Res. 18, 189–204. doi: 10.1007/BF02361722

[B42] Midmore D. J. Roca J. (1992). Influence of production and storage conditions on subsequent growth and tuber yield of potato (*Solanum* spp.) in the hot tropics. J. Agric. Sci. 119, 45–58. doi: 10.1017/S0021859600071537

[B43] Miller J. C. Scheuring D. C. Koym J. W. Holm D. G. (2005). TX1523-1Ru/Y a.k.a Sierra Gold™: An early maturing, yellow-fleshed russet cultivar for the specialty/gourmet market. Am. J. Potato Res. 82, 369–377. doi: 10.1007/BF02871967

[B44] Miller J. C. Scheuring D. C. Koym J. W. Holm D. G. Pavek J. J. Novy R. G.. (2018). Reveille Russet: An Early, Widely Adapted, high-count-carton russet for the fresh market. Am. J. Potato Res. 95, 79–86. doi: 10.1007/s12230-017-9620-2

[B45] Miller J. C. Scheuring D. C. Miller J. P. Fernandez G. C. J. (1999). Selection, evaluation, and identification of improved Russet Norkotah strains. Am. J. Potato Res. 76, 161–167. doi: 10.1007/BF02853581

[B46] Minhas J. S. Kumar D. Joseph T. A. Raj B. T. Khurana S. M. P. Pandey S. K.. (2006). Kufri Surya: A new heat tolerant potato variety suitable for early planting in North-Western plains, Peninsular India and processing into french fries and chips. Potato J. 33, 35–43.

[B47] Monneveux P. Ramírez D. A. Khan M. A. Raymundo R. M. Loayza H. Quiroz R. (2014). Drought and heat tolerance evaluation in potato (*Solanum tuberosum* L.). Potato Res. 57, 225–247. doi: 10.1007/s11540-014-9263-3

[B48] Morpurgo R. Ortiz R. (1988). Morphological variation of the potato(Solanum spp.) under contrasting environments. Environ. Exp. Bot. 28, 165–169. doi: 10.1016/0098-8472(88)90025-1

[B49] Muñoa L. Chacaltana C. Sosa P. Gastelo M. zum Felde T. Burgos G (2022). Effect of environment and peeling in the glycoalkaloid concentration of disease-resistant and heat-tolerant potato clones. J. Agric. Food Res. 7doi: 10.1016/j.jafr.2022.100269

[B50] Naz S. Ahmad S. Abbas G. Fatima Z. Hussain S. Ahmed M.. (2022). Modeling the impact of climate warming on potato phenology. Eur. J. Agron. 132, 126404. doi: 10.1016/j.eja.2021.126404

[B51] Nzaramba M. N. Scheuring D. C. Koym J. W. Miller J. C. (2013). Relationships among antioxidant activity, total phenolic content and specific gravity in several potato (*Solanum tuberosum* L.) cultivars grown in different environments. Am. J. Potato Res. 90, 541–550. doi: 10.1007/s12230-013-9326-z

[B52] Obiero C. O. Milroy S. P. Bell R. W. (2019). Importance of whole plant dry matter dynamics for potato (*Solanum tuberosum* L.) tuber yield response to an episode of high temperature. Environ. Exp. Bot. 162, 560–571. doi: 10.1016/j.envexpbot.2019.04.001

[B53] Obiero C. O. Milroy S. P. Bell R. W. (2022). Increasing frequency of high-temperature episodes in potato growing regions of Western Australia and its impacts on plant and tuber growth. Arch. Agron. Soil Sci. 68, 1988–2004. doi: 10.1080/03650340.2021.1948018

[B54] Patino-Torres A. J. Andrade M. H. M. L. Guedes M. L. Cavallin I. C. Pinto C. A. B. P. Souza J. C.. (2021). Performance of superior potato clones under high and mild temperatures in tropical climate. Agron. J. 113, 2349–2360. doi: 10.1002/agj2.20704

[B55] Paul S. Farooq M. Bhattacharya S. S. Gogoi N. (2017). Management strategies for sustainable yield of potato crop under high temperature. Arch. Agron. Soil Sci. 63, 276–287. doi: 10.1080/03650340.2016.1204542

[B56] Pradel W. Gatto M. Hareau G. Pandey S. K. Bhardway V. (2019). Adoption of potato varieties and their role for climate change adaptation in India. Clim. Risk Manage. 23, 114–123. doi: 10.1016/j.crm.2019.01.001 PMC7729832 33344151

[B1000] R Core Team. (2020). R: A language and environment for statistical computing. (Vienna, Austria: R Foundation for Statistical Computing) Available at: https://www.R-project.org/.

[B57] Raymundo R. Asseng S. Robertson R. Petsakos A. Hoogenboom G. Quiroz R.. (2018). Climate change impact on global potato production. Eur. J. Agron. 100, 87–98. doi: 10.1016/j.eja.2017.11.008

[B58] Rykaczewska K. (2013). The impact of high temperature during growing season on potato cultivars with different response to environmental stresses. Am. J. Plant Sci. 04, 2386–2393. doi: 10.4236/ajps.2013.412295

[B59] Rykaczewska K. (2015). The effect of high temperature occurring in subsequent stages of plant development on potato yield and tuber physiological defects. Am. J. Potato Res. 92, 339–349. doi: 10.1007/s12230-015-9436-x

[B60] Sonnewald S. Sonnewald U. (2014). Regulation of potato tuber sprouting. Planta 239, 27–38. doi: 10.1007/s00425-013-1968-z 24100410

[B61] Sood S. Bhardwaj V. Kumar V. Gupta V. K. (2020). BLUP and stability analysis of multi-environment trials of potato varieties in sub-tropical Indian conditions. Heliyon 6, e05525. doi: 10.1016/j.heliyon.2020.e05525 33294675 PMC7701190

[B62] Struik P. C. (2007). “Responses of the potato plant to temperature,” in Potato biology and biotechnology: advances and perspectives. Eds. Vreugdenhil D. Bradshaw J. Gebhardt C. Govers F. MacKerron D. K. L. Taylor M. A. (Kidlington, Oxford, UK: Elsevier B.V), 367–393. doi: 10.1016/B978-044451018-1/50060-9

[B63] Suttle J. C. (2007). “Dormancy and sprouting,” in Potato biology and biotechnology: advances and perspectives. Eds. Vreugdenhil D. Bradshaw J. Gebhardt C. Govers F. MacKerron D. K. L. Taylor M. A. (Kidlington, Oxford, UK: Elsevier), 287–309. doi: 10.1016/B978-0-444-51018-1.X5040-4

[B64] Tang R. Niu S. Zhang G. Chen G. Haroon M. Yang Q.. (2018). Physiological and growth responses of potato cultivars to heat stress. Botany 96, 897–912. doi: 10.1139/cjb-2018-0125

[B65] Teixeira A. L. Pinto C. A. B. P. Lepre A. L. Peixouto L. S. Ribeiro G. H. M. R. (2015). Evaluation of potato clones for heat tolerance in the southern region of Minas Gerais, Brazil. Rev. Bras. Ciências Agrárias - Braz. J. Agric. Sci. 10, 171–177. doi: 10.5039/agraria.v10i2a3268

[B66] Tilman D. Balzer C. Hill J. Befort B. L. (2011). Global food demand and the sustainable intensification of agriculture. Proc. Natl. Acad. Sci. 108, 20260–20264. doi: 10.1073/pnas.1116437108 22106295 PMC3250154

[B67] Timlin D. Lutfor Rahman S. M. Baker J. Reddy V. R. Fleisher D. Quebedeaux B. (2006). Whole plant photosynthesis, development, and carbon partitioning in potato as a function of temperature. Agron. J. 98, 1195–1203. doi: 10.2134/agronj2005.0260

[B68] Tito R. Vasconcelos H. L. Feeley K. J. (2018). Global climate change increases risk of crop yield losses and food insecurity in the tropical Andes. Glob. Change Biol. 24, e592–e602. doi: 10.1111/gcb.13959 29055170

[B69] United Nations. (2017). World population prospects: the 2017 revision, key findings and advance tables. Available online at: https://desapublications.un.org/publications/world-population-prospects-2017-revision.

[B70] Vales I. Miller C. Koym J. Scheuring D. (2020) Texas potato breeding report 2020. Available online at: http://potato.tamu.edu/reports.html.

[B71] Vales M. I. Scheuring D. C. Koym J. W. Holm D. G. Essah S. Y. C. Wilson R. G.. (2022). Vanguard russet: A fresh market potato cultivar with medium-early maturity and long dormancy. Am. J. Potato Res. 99, 258–267. doi: 10.1007/s12230-022-09877-0

[B72] Van Dam J. Kooman P. L. Struik P. C. (1996). Effects of temperature and photoperiod on early growth and final number of tubers in potato (*Solanum tuberosum* L.). Potato Res. 39, 51–62. doi: 10.1007/BF02358206

[B73] Van Den Berg J. H. Struik P. C. Ewing E. E. (1990). One-leaf cuttings as a model to study second growth in the potato (*Solanum tuberosum* ) plant. Ann. Appl. Biol. 66, 273–280. doi: 10.1093/oxfordjournals.aob.a088025

[B74] Van Ittersum M. K. Aben F. C. B. Keijzer C. J. (1992). Morphological changes in tuber buds during dormancy and initial sprout growth of seed potatoes. Potato Res. 35, 249–260. doi: 10.1007/BF02357705

[B75] Webb R. E. Wilson D. R. Shumaker J. R. Graves B. Henninger M. R. Watts J.. (1978). Atlantic: A new potato variety with high solids, good processing quality, and resistance to pests. Am. Potato J. 55, 141–145. doi: 10.1007/BF02852087

[B76] Wheeler R. M. Tibbitts T. W. (1986). Growth and tuberization of potato (*Solanum tuberosum* L.) under continuous light. Plant Physiol. 80, 801–804. doi: 10.1104/pp.80.3.801 11539039 PMC1075206

[B77] Wolf S. Marani A. Rudich J. (1990). Effects of temperature and photoperiod on assimilate partitioning in potato plants. Ann. Bot. 66, 513–520. doi: 10.1093/oxfordjournals.aob.a088060

[B78] Wurr D. C. E. (1992). “Seed tuber production and management,” in The potato crop. Ed. Harris P. (Springer Netherlands, Dordrecht), 1–12. doi: 10.1007/978-94-011-2340-2_1

[B79] Yamori W. Hikosaka K. Way D. A. (2014). Temperature response of photosynthesis in C3, C4, and CAM plants: temperature acclimation and temperature adaptation. Photosynth. Res. 119, 101–117. doi: 10.1007/s11120-013-9874-6 23801171

[B80] Yandell B. S. Najar A. Wheeler R. Tibbitts T. W. (1988). Modeling the effects of light, carbon dioxide, and temperature on the growth of potato. Crop Sci. 28, 811–818. doi: 10.2135/cropsci1988.0011183X002800050019x 11539763

[B81] Yencho G. C. McCord P. H. Haynes K. G. Sterrett S. B. R. (2008). Internal heat necrosis of potato—A review. Am. J. Potato Res. 85, 69–76. doi: 10.1007/s12230-008-9008-4

[B82] Zhang G. Tang R. Niu S. Si H. Yang Q. Rajora O. P.. (2021). Heat-stress-induced sprouting and differential gene expression in growing potato tubers: Comparative transcriptomics with that induced by postharvest sprouting. Hortic. Res. 8, 226. doi: 10.1038/s41438-021-00680-2 34654802 PMC8519922

[B83] Zhao C. Stockle C. O. Karimi T. Nelson R. L. van Evert F. K. Pronk A. A.. (2022). Potential benefits of climate change for potatoes in the United States. Environ. Res. Lett. 17. doi: 10.1088/1748-9326/ac9242

[B84] Zommick D. H. Knowles L. O. Pavek M. J. Knowles N. R. (2014). In-season heat stress compromises postharvest quality and low-temperature sweetening resistance in potato (*Solanum tuberosum* L.). Planta 239, 1243–1263. doi: 10.1007/s00425-014-2048-8 24615233

