# Supplementary material for: Assessing heat tolerance in potatoes: Responses to stressful Texas field locations and controlled contrasting greenhouse conditions
Source: Front Plant Sci. 2024 May 13;15:1364244. doi: 10.3389/fpls.2024.1364244 (PMC11128680; doi:10.3389/fpls.2024.1364244)
Supplement: Supplementary file 1 [file DataSheet_1.docx]

**Supplementary files**

**SUPPLEMENTARY FIGURE 1.** Day length in Dalhart and Springlake, Texas, during potato growing seasons 2019-2021. (Data source: <https://gml.noaa.gov/>)

**SUPPLEMENTARY FIGURE 2|** Phenotypic variance explained for yield and quality traits in potatoes grown in two locations (Springlake and Dalhart, TX) for three years (2019, 2020, and 2021). TY – total yield (Mg/ha), US – marketable yield (Mg/ha), PID – percent tubers affected with internal defects, ATN – average tuber number per plant, ATW – average tuber weight (g), SG – specific gravity, DOR – dormancy (days). * Due to the unbalanced dataset (missing plots), the sum of squares due to individual sources of variation on some traits did not amount to the total sum of squares.

**SUPPLEMENTARY FIGURE 3|** Phenotypic variance explained for yield and quality traits in potatoes grown on two different planting dates (regular *vs.* late) in Springlake, TX, in 2019 and 2020. * Due to the unbalanced dataset (missing plots), the sum of squares due to individual sources of variation on some traits did not amount to the total sum of squares.

**SUPPLEMENTARY FIGURE 4|** Phenotypic variance explained for yield and quality traits in potatoes grown under two different temperature conditions (normal *vs*. heat stress) in greenhouses for two years (2020 and 2021). PH – plant height (cm), LAI – leaf area (cm^2^), ADM – aerial dry matter(g), TY– total yield (g/plant), US – marketable yield (g/plant), CU – culls yield (g/plant), PTED – percent tubers affected with external defects, PID – percent tubers affected with internal defects, ATN – average tuber number per plant, ATW – average tuber weight (g), SG – specific gravity, HI – harvest index, DOR – dormancy (days after vine kill)


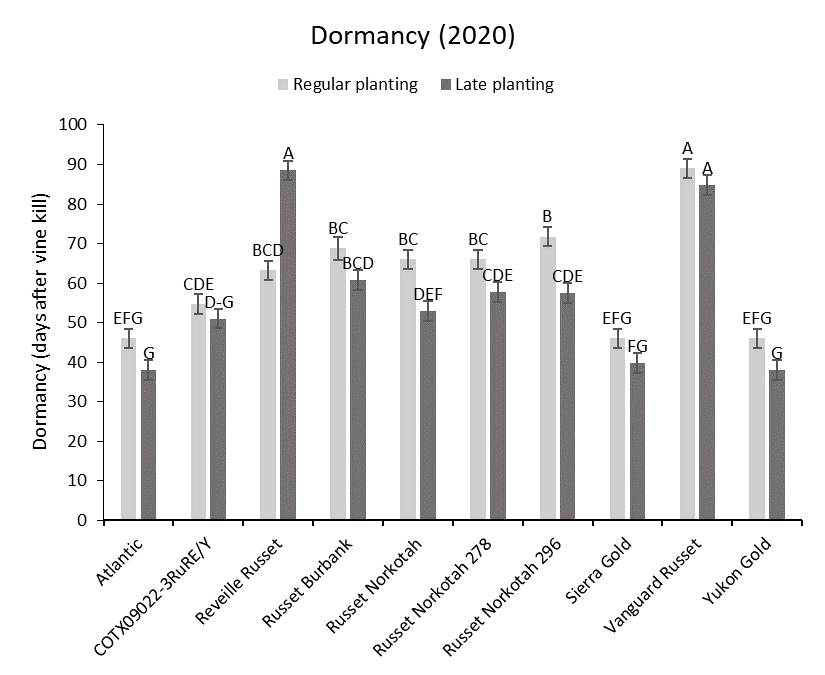


**SUPPLEMENTARY FIGURE 5|** Means (least squares) of dormancy in ten potato clones grown at two different planting times (normal and late) in Springlake, TX, in 2020. Bars that do not share a letter are significantly different at p< 0.05 (Tukey’s HSD).

**SUPPLEMENTARY FIGURE 6|** Relationship and classification of environments for different selected traits measured in two potato growing sites of Texas (Springlake and Dalhart) in 2019, 2020 and 2021. TY - total yield (Mg/ha), PUS - percent marketable tubers, PCU - percent cull tubers, PID - percent tubers affected with internal defects, ATN - average tuber number per plant, ATW - average tuber weight.


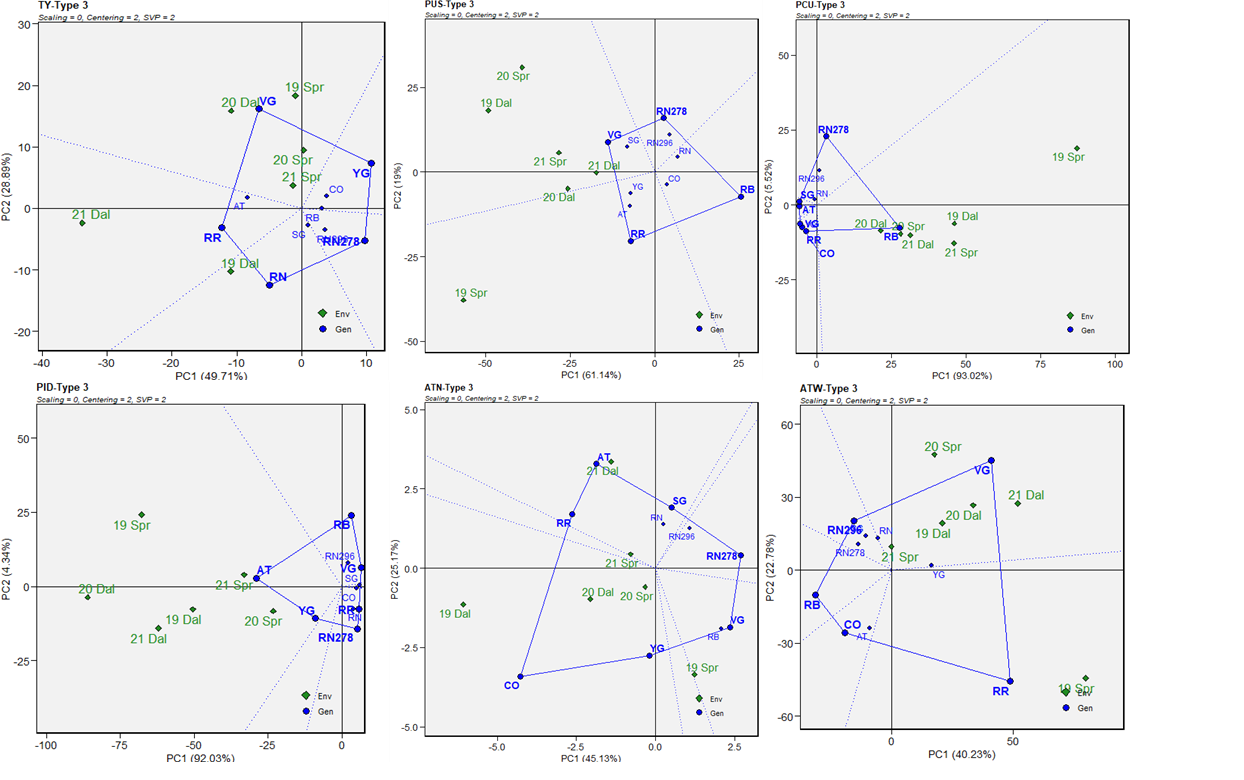


**SUPPLEMENTARY FIGURE 7|** Which-won-where view of the GGE biplot for selected traits measured in two potato growing sites of Texas (Springlake and Dalhart) in 2019, 2020 and 2021. TY - total yield (Mg/ha), PUS - percent marketable tubers, PCU - percent cull tubers, PID - percent tubers affected with internal defects, ATN - average tuber number per plant, ATW - average tuber weight. AT (Atlantic), CO (COTX09022-3RuRE/Y), RR (Reveille Russet), RB (Russet Burbank), RN (Russet Norkotah), RN278 (Russet Norkotah 278), RN296 (Russet Norkotah 296), Sierra Gold (SG), Vanguard Russet (VR) and Yukon Gold (YG)

**SUPPLEMENTARY TABLE 1|** Planting and harvest dates of ten clones tested at two locations (Dalhart and Springfield) in Texas from 2019-2021.

|  | **Springlake** | | |  |  | **Dalhart** | | |  |
| --- | --- | --- | --- | --- | --- | --- | --- | --- | --- |
| **Year** | **Planting Date** | **Harvesting Date** | **Crop Length** | **Spacing** |  | **Planting Date** | **Harvesting Date** | **Crop Length** | **Spacing** |
|  |  |  | **Days** | **cm *cm** |  |  |  | **Days** | **cm *cm** |
| 2019 | 23-Mar | 11-Jul | 110 | 91.4*22.9 |  | 13-May | 20-Sep | 130 | 71.1*23.0 |
| 2020 | 28-Mar | 12-Jul | 106 | 91.4*22.9 |  | 11-May | 3-Sep | 116 | 71.1*23.0 |
| 2021 | 31-Mar | 29-Jul | 121 | 91.4*22.9 |  | 5-May | 15-Sep | 134 | 71.1*23.0 |

**SUPPLEMENTARY TABLE 2|** Planting and harvest dates of ten clones tested at two different planting dates in Springlake, Texas, for 2019 and 2020.

| **Year** | **Regular planting** | | |  | **Late planting** | | |
| --- | --- | --- | --- | --- | --- | --- | --- |
|  | **Planting date** | **Harvesting date** | **Crop Length** |  | **Planting date** | **Harvesting date** | **Crop Length** |
|  |  |  | **Days** |  |  |  | **Days** |
| 2019 | 23-Mar | 11-Jul | 110 |  | 17-May | 30-Aug | 106 |
| 2020 | 28-Mar | 12-Jul | 106 |  | 5-May | 16-Aug | 103 |

**SUPPLEMENTARY TABLE 3|** Planting and harvest dates of ten clones tested at two different temperature conditions in greenhouses for 2020 and 2021.

| **Year** | **Normal** | | |  | **Heat stress** | | |
| --- | --- | --- | --- | --- | --- | --- | --- |
|  | **Planting date** | **Harvesting date** | **Crop Length** |  | **Planting date** | **Harvesting date** | **Crop Length** |
|  |  |  | **Days** |  |  |  | **Days** |
| 2020 | 24-Feb | 27-May | 92 |  | 17-May | 27-May | 92 |
| 2021 | 24-Feb | 3-Jun | 99 |  | 5-May | 3-Jun | 99 |

**SUPPLEMENTARY TABLE 4|** Test for fixed effects of location, clone and their interaction on tuber yield and quality traits of potato grown in Springlake and Dalhart, Texas, during 2019-2021.

| **Sources of Variation** | **Total yield** | **Marketable yield** | **Percent tubers with internal defects** | **Average tuber number** | **Average tuber weight** | **Specific gravity** | **Dormancy** |
| --- | --- | --- | --- | --- | --- | --- | --- |
|  | **Mg/ha** | **Mg/ha** | **%** | **no/plant** | **g/tuber** |  | **Days** |
| **2019** |  |  |  |  |  |  |  |
| Location | *** | *** | ns | *** | *** | *** |  |
| Clone | ns | *** | *** | *** | *** | *** |  |
| Location*Clone | ns | ** | * | *** | *** | *** |  |
| **2020** |  |  |  |  |  |  |  |
| Location | *** | *** | ** | ns | *** | ns | *** |
| Clone | *** | *** | *** | ns | *** | *** | *** |
| Location*Clone | * | *** | *** | ns | *** | ns | *** |
| **2021** |  |  |  |  |  |  |  |
| Location | *** | *** | *** | *** | *** | ns | *** |
| Clone | *** | *** | *** | *** | *** | *** | *** |
| Location*Clone | *** | *** | ns | *** | *** | * | *** |

ns- non-significant, * p< 0.05, ** p< 0.01, *** p< 0.001.

**SUPPLEMENTARY TABLE 5|** Test for fixed effects of planting dates, clone and their interaction on yield and quality traits of potatoes grown in Springlake, Texas during 2019 and 2020.

| **Sources of Variation** | **Total yield** | **Marketable yield** | **Percent tubers with Internal defects** | **Average tuber number** | **Average tuber weight** | **Specific gravity** | **Dormancy** **ˆ** |
| --- | --- | --- | --- | --- | --- | --- | --- |
|  | **Mg/ha** | **Mg/ha** | **%** | **no/plant** | **g/tuber** |  | **Days** |
| Planting Date | ns | ns | ns | ns | ns | ns | *** |
| Clone | ns | * | * | * | ns | * | *** |
| Planting Date*Clone name | ns | ns | ns | ns | ns | ** | *** |

ns non-significant, * p< 0.05, ** p< 0.01, *** p< 0.001. **ˆ** for year 2020 only.

**SUPPLEMENTARY TABLE 6|** Test for fixed effects of temperature conditions, clone and their interaction on yield and quality traits of ten potatoes grown in greenhouses in Texas in 2020 and 2021.

| **Sources of Variation** | **Plant height** | **Leaf area** | **Aerial dry matter** | **Total yield/plant** | **Marketable yield/plant** | **Percent tubers with external defects** | **Percent tubers with internal defects** | **Average tuber number** | **Average Tuber Weight** | **Specific gravity** | **Harvest index** | **Dormancy** |
| --- | --- | --- | --- | --- | --- | --- | --- | --- | --- | --- | --- | --- |
|  | **cm** | **cm^2^** | **g** | **g** | **g** | **%** | **%** | **no/plant** | **g/tuber** |  |  | **Days** |
| **2020** |  |  |  |  |  |  |  |  |  |  |  |  |
| Clone | *** |  | *** | ns | *** | ** | *** | *** | *** | *** | *** | *** |
| Condition | *** |  | *** | ns | ns | ns | ns | ns | ns | *** | *** | ns |
| Clone*Condition | * |  | * | * | * | * | *** | ** | * | ** | *** | ns |
| **2021** |  |  |  |  |  |  |  |  |  |  |  |  |
| Clone | *** | *** | *** | *** | *** | *** | *** | *** | * | *** | *** | *** |
| Condition | *** | *** | *** | * | ** | * | *** | ** | ** | *** | *** | ** |
| Clone*Condition | ns | ns | ns | * | ** | *** | *** | ** | * | * | ns | ns |

ns non-significant, * p< 0.05, ** p< 0.01, *** p< 0.001.

**SUPPLEMENTARY TABLE 7|** Least squares means of ten potato clones evaluated under greenhouse conditions in 2020 to assess the effect of different temperature conditions (normal *vs.* heat stress) on some selected traits.

| **Clone** | **2020** | | |  | **2021** | | | |
| --- | --- | --- | --- | --- | --- | --- | --- | --- |
|  | **Plant height** | **Aerial dry matter** | **Harvest index** |  | **Plant height** | **Aerial dry matter** | **Leaf area** | **Harvest index** |
|  | **cm** | **g/plant** |  |  | **cm** | **g/plant** | **cm^2^** |  |
| **Normal** |  |  |  |  |  |  |  |  |
| Atlantic | 69.9g | 31.6e-i | 0.85 a |  | 86.3g | 43.8def | 176.2abc | 0.86 ab |
| COTX09022-3RuRE/Y | 78.9fg | 29.8f-i | 0.84 abc |  | 107.7b-g | 35.8ef | 95.3c-f | 0.82 ab |
| Reveille Russet | 88.5d-g | 32.8e-i | 0.81 a-d |  | 100.0d-g | 49.3c-f | 179.6ab | 0.73 a-d |
| Russet Burbank | 108.3bcd | 39.1c-g | 0.83 a-d |  | 140.8ab | 57.1b-e | 118.7bcd | 0.83 ab |
| Russet Norkotah | 69.3g | 25.3hi | 0.86 a |  | 92.3fg | 33.1ef | 174.7abc | 0.84 ab |
| Russet Norkotah 278 | 72.1fg | 27.7ghi | 0.86 a |  | 111.4b-g | 42.6def | 120.1bc | 0.79abc |
| Russet Norkotah 296 | 77.8fg | 29.9f-i | 0.85 abc |  | 98.1efg | 35.1ef | 141.3abc | 0.84 ab |
| Sierra Gold | 73.4fg | 30.0f-i | 0.85 a |  | 101.8c-g | 48.3c-f | 185.0ab | 0.85 ab |
| Vanguard Russet | 89.9c-g | 23.7i | 0.83 a-d |  | 85.7g | 37.5ef | 183.5ab | 0.87 a |
| Yukon Gold | 80.8efg | 31.3e-i | 0.85 ab |  | 94.5efg | 30.2f | 208.6a | 0.89 a |
| ***Average Normal*** | *80.9* | *30.1* | *0.84* |  | *101.9* | *41.3* | *158.3* | *0.83* |
|  |  |  |  |  |  |  |  |  |
| **Heated** |  |  |  |  |  |  |  |  |
| Atlantic | 79.2fg | 46.0bcd | 0.77 cde |  | 98.2efg | 66.6a-d | 56.7de | 0.72a-d |
| COTX09022-3RuRE/Y | 95.8b-f | 35.1d-i | 0.81 a-d |  | 129.5b-e | 64.4a-d | 31.3f | 0.72a-d |
| Reveille Russet | 118.9b | 53.0b | 0.71 ef |  | 143.5ab | 66.0a-d | 38.0ef | 0.51e |
| Russet Burbank | 147.8a | 67.9a | 0.66 f |  | 172.3a | 87.5a | 22.9f | 0.60cde |
| Russet Norkotah | 96.0b-f | 39.8c-g | 0.79 a-d |  | 126.5b-f | 63.4a-d | 36.1f | 0.67b-e |
| Russet Norkotah 278 | 110.1bcd | 46.9bcd | 0.77 de |  | 141.3ab | 78.7ab | 28.6f | 0.52e |
| Russet Norkotah 296 | 115.1bc | 48.3bc | 0.76 de |  | 137.4abc | 77.8ab | 29.1f | 0.56de |
| Sierra Gold | 95.8b-f | 43.8b-e | 0.78 b-e |  | 136.5a-d | 69.9abc | 48.9def | 0.75a-d |
| Vanguard Russet | 105.8b-e | 37.7c-h | 0.81 a-d |  | 102.2c-g | 51.5c-f | 96.5c-f | 0.74a-d |
| Yukon Gold | 107.3bcd | 42.5b-f | 0.82 a-d |  | 139.1ab | 64.1a-d | 45.4def | 0.78abc |
| ***Average Heated*** | *107.2* | *46.1* | *0.77* |  | *132.7* | *69.0* | *43.4* | *0.66* |
| ***Change (***%) | ***32.5*** | ***53.2*** | **-8.3** |  | ***30.2*** | ***67.1*** | **-72.6** | **-20.5** |

Means followed by the same letter in the same column were not significantly different by Tukey’s HSD test at the 5% significance level. Negative values indicate a decrease, and positive values indicate an increase in trait values from the normal to the heated condition.
